# Methylation profiling and alternative classification approaches in a glioma-enriched FFPE stereotaxic biopsy cohort

**DOI:** 10.1186/s40478-026-02382-z

**Published:** 2026-07-22

**Authors:** Katharina Johanna Weber, Jürgen Hench, Markus Zumpt, Thomas Eska, Moritz Armbrust, Pia Susann Zeiner, Tabea Isabelle Hartung, Viktoria Ruf, Eike Steidl, Mareike Dettki, Tim Fenton, Joachim Peter Steinbach, Marcus Czabanka, David Capper, Stephan Frank, Karl-Heinz Plate, Marie-Thérèse Forster, Patrick Nikolaus Harter

**Affiliations:** 1 Goethe University Frankfurt, University Hospital, Neurological Institute (Edinger Institute), Heinrich-Hoffmann-Straße 7, 60528 Frankfurt am Main, Germany; 2 German Cancer Research Center (DKFZ), German Cancer Consortium (DKTK), Partner Site Frankfurt, Im Neuenheimer Feld 280, 69120 Heidelberg, Germany; 3 Frankfurt Cancer Institute (FCI), Paul-Ehrlich-Str. 42-44, 60596 Frankfurt am Main, Germany; 4 Goethe University Frankfurt, University Hospital, University Cancer Center (UCT), Theodor-Stern-Kai 7, 60590 Frankfurt am Main, Germany; 5 Division of Neuropathology, Institute of Pathology, Basel University Hospital, Schönbeinstrasse 40, 4031 Basel, Switzerland; 6 Ludwig Maximilians University Munich, University Hospital, Center for Neuropathology and Prion Research, Faculty of Medicine, Feodor-Lynen-Straße 23, 81377 Munich, Germany; 7 German Cancer Consortium (DKTK), Partner Site Munich, A Partnership Between German CancerResearch Center (DKFZ) and University/University Hospital, Ludwig Maximilians University Munich, Marchioninistraße 15, 81377 Munich, Germany; 8 Goethe University Frankfurt, University Hospital, Dr. Senckenberg Institute of Neurooncology, Theodor-Stern-Kai 7, 60590 Frankfurt am Main, Germany; 9 Goethe University Frankfurt, University Hospital, Department of Neurology, Theodor-Stern-Kai 7, 60590 Frankfurt am Main, Germany; 10 University Hospital Hamburg-Eppendorf, Martinistraße 52, 20251 Hamburg, Germany; 11 Goethe University Frankfurt, University Hospital, Institute of Neuroradiology, Theodor-Stern-Kai 7, 60590 Frankfurt am Main, Germany; 12 School of Cancer Sciences, University of Southampton, University Road, Southampton, SO17 1BJ UK; 13 Goethe University Frankfurt, University Hospital, Department of Neurosurgery, Theodor-Stern-Kai 7, 60590 Frankfurt am Main, Germany; 14 Charité - Universitätsmedizin Berlin, Corporate Member of Freie Universität Berlin and Humboldt-Universität zu Berlin, Department of Neuropathology, Charitéplatz 1, 10117 Berlin, Germany; 15 German Cancer Consortium (DKTK), Partner Site Berlin and German Cancer Research Center (DKFZ), Im Neuenheimer Feld 280, 69120 Heidelberg, Germany; 16 Bavarian Center for Cancer Research (BZKF), Partner Site Munich, Marchioninistraße 15, 81377 Munich, Germany

**Keywords:** Glioblastoma, IDH-wildtype, Heidelberg brain tumor classifier, Bethesda brain tumor classifier, EpiDiP, Tumor deconvolution, Stereotaxic biopsy

## Abstract

DNA methylation profiling of CNS tumors supports and refines histological diagnoses. Tumor DNA methylation signatures allow for an alignment with reference methylation classes and copy number profiling required for molecular grading. Overall, and especially in cases with limited tissue like in stereotaxic (STX) biopsies, there is a need for informed triage to most promising analysis techniques. Here, we investigated the diagnostic potential and accuracy of DNA methylation profiling and molecular grading in STX samples. FFPE samples of 237 patients were subjected to DNA methylation analysis including the Heidelberg (v11b4), Bethesda (v2) and EpiDiP brain tumor classifiers, copy number profiling and tumor deconvolution using MethylCIBERSORT. 90.5% of samples (*n* = 232) were allocated DNA methylation classes, with 61.6% reaching Heidelberg classifier scores of > 0.84. Indicatory copy number variations (CNVs) were detectable in 96.9% of cases. Recursive partitioning supported by a resampling-based validation with repeated random subsampling pointed towards brain tumor classifier dependent minimum analyte inputs to reach robust calibrated scores for DNA methylation class allocation. Successful integrative diagnostics furthermore depended on pre-classifier H&E diagnoses with 96.2% of H&E malignant diffuse gliomas, 91.5% of H&E diffuse gliomas but only 60% of H&E circumscribed gliomas classifiable using the Heidelberg brain tumor classifier. When the alternative classifiers EpiDiP and Bethesda were used, higher DNA input amounts ameliorated the classification robustness. Nevertheless, diagnostics of high-grade gliomas in midline location and lower-grade glial/glioneuronal tumors posed a challenge. Our findings demonstrate the broad applicability of DNA methylation profiling and molecular grading to small brain tumor specimens under the caveat of certain tumors and locations potentially benefiting from additional molecular as well as computational approaches to increase methylation signals relevant for classification in the future.

**Supplementary Information:**

The online version contains supplementary material available at 10.1186/s40478-026-02382-z.

## Introduction

Central nervous system tumors affect patients across all age groups with average annual age-adjusted incidence rates per 100 000 US citizens of 5.83, 11.54, and 42.85 for age groups 0–14, 15–39 and above 40 years, respectively [24]. Patient outcome is mainly determined by tumor type and CNS WHO grade [20, 24]. Additionally, tumor location and growth patterns determine patient outcome too, since they impact feasibility of gross resection [5]. In these cases, stereotaxic biopsy (STX) is the method of choice to obtain samples for further neuropathological analyses. However, STX samples usually measure only a few millimeters in size, which may limit the range of analysis techniques.

DNA methylation-based CNS tumor classification changed neuropathological routine work-up tremendously by adding an independent diagnostic level to histology and genetics [6, 17]. As robust tumor-specific epigenetic phenomenon, DNA methylation continues to contribute to refined subgrouping of brain tumor entities and is thus comprehensively implemented in diagnostics [4, 17, 32, 33]. Genome-wide DNA methylation analysis allows for the profiling of relevant copy number alterations on chromosome and gene locus level as defined by the 5th edition of the WHO Classification of Central Nervous System Tumours [20]. Simultaneously, methylation data can readily be aligned with reference data sets within the frame of machine learning-based tumor classification algorithms. A growing number of brain tumor classifiers reflect the evolving understanding of tumor methylation phenotypes and their clinical significance, paving the way for new tumor subtype definition [31, 38].

State-of-the-art neuropathological diagnostics aim for the integration of histological and molecular tumor characteristics which at best match and complement each other. Precise diagnoses pioneer treatment regimen [14]. On the contrary, failure of classification hampers clinical decision making, as exemplified by the prolonged time until treatment observed for patients whose tumors rendered calibrated scores < 0.3 in methylation profiling [9]. The reasons for non-matching classifier scores beneath 0.3 are manifold and can relate to low proportions of tumor cells within a DNA isolate or an overall low DNA amount. Prior studies raised controversial results about the parameter of low input DNA on the assignability of a matching classifier score [9, 38]. The influence of a limited sample size itself on confident classifier results as well as copy number profiles remains unexplored.

While molecular grading and the application of molecular analysis techniques in modern brain tumor diagnostics become increasingly important, technical guidelines for the assessment of alterations, like *TERT* promoter mutations, copy number variations or *EGFR* amplification are not strictly outlined in the WHO classification [20]. Detailed information about optimal sample sizes including cancer cell proportions or DNA concentration needed for proper molecular pathological assessment of brain tumors are not specified or subject to standardization efforts, either. Even though some specifications are decreed by the assay providers and users, issues regarding the usability of low sample DNA inputs for diagnostic purposes remain less well studied [28].

Therefore, we set out to retrospectively examine the utility of primary brain tumor samples obtained by STX for profound DNA methylation-based neuropathological diagnostics and to elucidate the question of lowest DNA and cancer cell thresholds using a real-life cohort.

## Materials and methods

### Patient selection and STX procedure

Patients undergoing STX with subsequent molecular pathological analysis between January 2017 and October 2020 at the Department of Neurosurgery of the University Hospital and the Neurological Institute (Edinger Institute) in Frankfurt, respectively, were enrolled in this retrospective study. All patients suffered from intracranial lesions. Patients were subjected to either frame-less (ROSA™ Robotic Stereotactic Assistant) or frame-based STX. Prior to the procedure, MRI scans were performed and trajectories with biopsy targets were calculated. The study was approved by the local ethical committee (SNO-19-2020).

### Intraoperative diagnostics

For intraoperative tissue evaluation a smear preparation was conducted by a neuropathologist (KJW, MA, PNH) and stained with methylene blue (Chroma 1B429, Merck, Darmstadt, Germany). The samples were inspected by use of the Olympus Microscope BX41 (Olympus Europe, Hamburg, Germany) inside the operating room.

### Tissue processing for histological evaluation

With the exception of tissue collected for smear preparation, all specimens obtained via STX were formalin-fixed and paraffin-embedded (FFPE) following common protocols. The sample size was 1 mm³ in average. 4 μm thin tissue sections were cut with a microtome (Leica SM 2000R, Wetzlar, Germany) and mounted on slides (Superfrost Plus, Thermo Scientific, Braunschweig, Germany). After H&E immunohistochemical staining was performed at least with mutation specific antibodies against IDH1_R132H (clone H09, Dianova, Hamburg, Germany) and Histone 3 Lysin 27 Methionine (H3K27M) (clone RM192, Dianova, Hamburg, Germany), as well as against ATRX (clone BSB-108, Bio SB, Santa Barbara, California, USA), Ki67 (clone MIB-1, Agilent Technologies, Inc., Santa Clara, California, USA) and H3K27me3 (clone C36B11, Cell Signaling Technology, Danvers, Massachusetts, USA) following established protocols by use of a LEICA BOND-III automated stainer (Leica, Wetzlar, Germany). Throughout the manuscript this procedure will be named “Routine diagnostic workup” (RDW). Tissue sections were evaluated by at least two experienced neuropathologists (KJW, MA, PNH). Representative, non-necrotic sections of the respective lesion were selected and subjected to further molecular analysis. Tumor classification was based on the 5th edition of the WHO Classification of Tumours of the Central Nervous System [20].

### Collection of large-scale DNA methylation data

Selected tissue samples were transferred into Eppendorf tubes by use of biopsy punches (1.5 mm diameter, kai Europe GmbH, Solingen, Germany). For DNA extraction the Stratek Invisorb Genomic DNA Kit II (stratek molecular, Berlin, Germany) and the Maxwell RSC FFPE Plus DNA Kit (Promega, Madison, Wisconsin, USA) were used following the manufacturer’s protocol. DNA concentration was measured by use of the Qubit DNA BR Assay Kit and Qubit 3 Fluorometer device (Invitrogen, Life Technologies Corporation, Oregon, USA). DNA was further processed and hybridized to the Human Methylation EPIC array beadchip (v.1; Illumina, San Diego, California, USA) following protocols provided by the manufacturer. An input of 250 ng DNA onto the beadchip was aimed for overall. In samples below a DNA concentration of 5.56 ng/µl, i.e. samples not reaching an absolute amount of 250 ng DNA, a maximal volume of 45 µl was used (Supplementary Excel File). EPIC array beadchips were scanned by an iScan (Illumina, San Diego, California, USA) and raw intensity data (idats) was obtained.

### Analysis of DNA methylation data

Idats were uploaded to the website molecularpathology.org provided by the University of Heidelberg, Germany until February 2024 to obtain calibrated scores for DNA methylation classes and subclasses, copy number variation profiles and MGMT promoter methylation status (© MolecularNeuroPathology.org 2018 - Version 3.1.5). The Heidelberg brain tumor classifier version “11b4” was used for this study. Copy number profiles were evaluated visually according to Shirahata et al. and Stichel et al. [30, 33]. In detail, gain of chromosome 7 and loss of chromosome 10 were determined visually by estimation of copy number variability over all chromosomes in relation to the respective chromosomes or gene locus of interest. For *EGFR* amplification an estimated cutoff of 0.35 was deemed significant. Homozygous deletion of *CDKN2A/B* was analyzed visually by comparison of the drop of the copy number profile as compared to the copy number variability according to [15]. The samples were analyzed using the brain tumor classifiers “Bethesda version 2” provided by the National Cancer Institute, Center for Cancer Research via the tool “Methylscape Analysis” and “EpiDiP” provided by the Divison of Neuropathology, Institute for Medical Genetics and Pathology, University Hospital Basel, Switzerland [13] using the reference set “AllIDATv2_20210804_HPAP_Sarc_NanoDiP”. The EpiDiP annotation „GBM_NOS“ refers to malignant diffuse gliomas without matching overlap with predefined subclasses and missing pathognomonic copy number profiles. Because of reported high-confidence allocation to methylation classes already achievable by use of a cut off of 0.84, we focused on this calibrated score for the Heidelberg classifier mainly [6]. Since comparable information is currently missing for the Bethesda classifier, the 0.9 cut off described as matching alignment of a sample to a methylation family/class was used. Additionally, idats were fed into the R software package “RnBeads” [22]. RnBeads uses built-in control probes on the EPIC array beadchip for quality control. Samples and/or Cytosine-phosphate-Guanine (CpG) probes not showing overall high quality using the iterative *Greedycut* algorithm implemented in *RnBeads*, were removed. Impure and therefore discarded samples were identified through beta value *p* values exceeding the 0.05 threshold [22]. Furthermore, CpGs were filtered according to detection p-values, and annotated SNPs, sites on the sex chromosomes and potentially cross-reactive sites were discarded from the analysis. Methylation data was normalized using the “dasen” method from the R package “wateRmelon”. RnBeads allows for computation of overall leukocyte contents, too, by the implemented “leukocyte unmethylation for purity” (LUMP) algorithm which also allows for tumor purity estimation. The leukocyte ratio is thereby inferred from the screening of DNA methylation data for 44 CpG sites particularly hypomethylated in leukocytes [2]. For tumor purity estimates we further applied the reference-based tumor deconvolution algorithm “MethylCIBERSORT”. MethylCIBERSORT allows for inferring proportions of tumor cells, immune cell subsets, neurons, and glia from bulk tumor DNA methylation data by comparison of sample methylomes to reference methylomes [7, 23]. MethylCIBERSORT analysis was carried out according to the protocol and reference methylomes were modified according to [7, 10]. Briefly, idats were imported into the R “minfi” package to perform quality checks, Noob normalization and acquisition of beta values. The sample beta value matrices and the reference file (for IDH-wildtype glioblastoma) were uploaded onto the CIBERSORTx website and deconvoluted (provided by the Alizadeh Lab, Stanford University, USA, developed by Newman et al. [23]).

### *TERT* promoter mutation analysis

*TERT* promoter status was analysed by amplifying the *TERT* promoter region including the two hot spot positions C228 and C250 located − 124 bp and − 146 bp upstream of the *TERT* gene, respectively (QIAGEN MultiplexPCR Kit, Qiagen, Venlo, Netherlands) followed by Sanger sequencing (SeqStudio Genetic Analyzer, Applied Biosystems by Thermo Fisher Scientific, Waltham, Massachusetts, USA).

### tSNE analysis

The idat files were filtered and normalized using the “pfilter” and “dasen” functions from the “wateRmelon” package (v2.10.0). Dimensionality Reduction was subsequently performed using the “Rtsne” package (v0.17) on the 10,000 most variable CpG sites measured by the methylation array. The results were visualized using “ggplot2” (v3.5.1). The analysis was conducted using R (v4.4.3).

### Statistical analysis and figure design

**Fig. 1:**
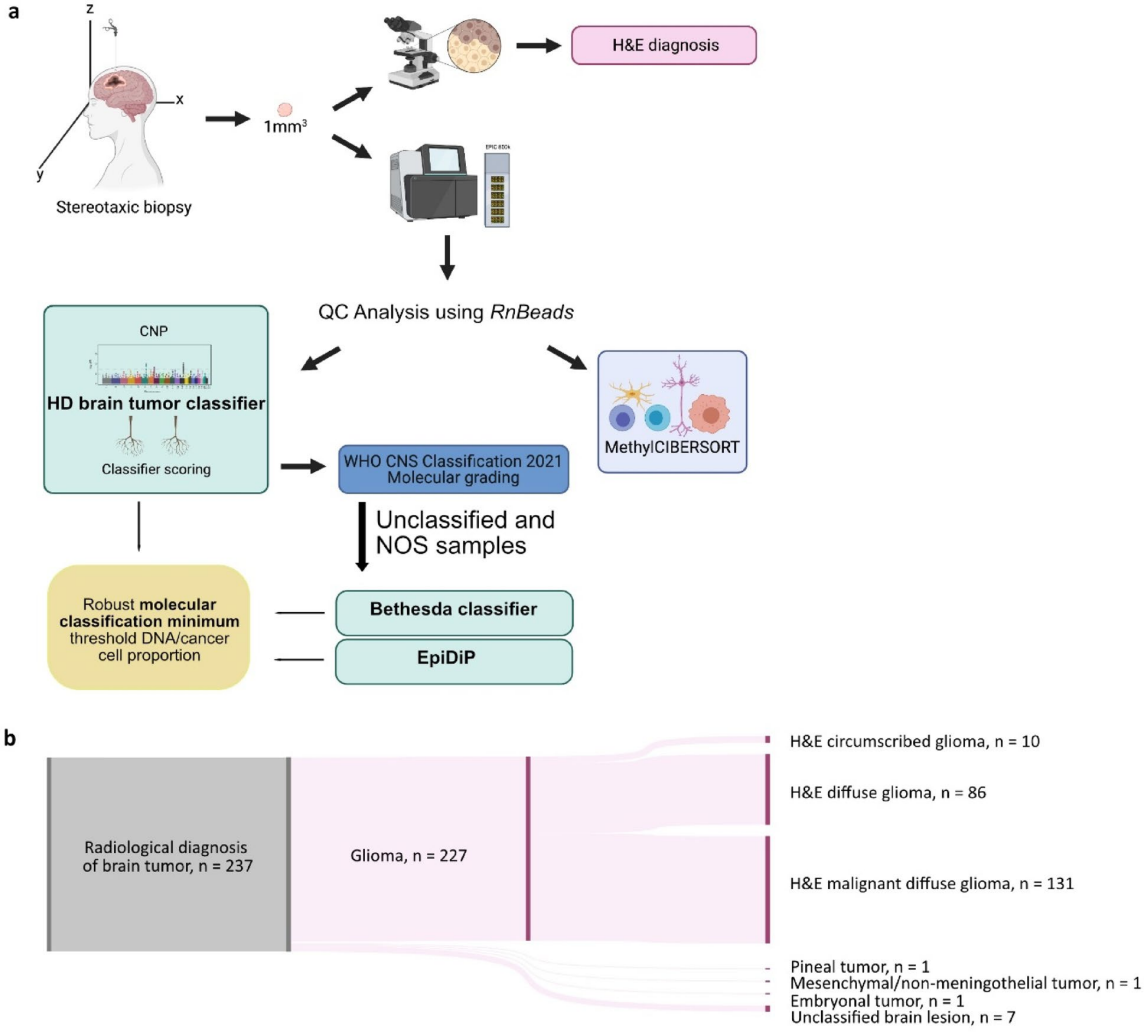
Study workflow and patient cohort. **a** Schematic overview of study workflow. Created with BioRender. **b** Total study cohort of 237 patients with brain tumors, stratified according to brain tumor type (“glioma” or others) and glioma types in detail, sankey plot

All statistical analyses, including the recursive portioning model with cancer cell proportions and DNA concentration as predictor of the dependent variable “calibrated score bigger vs. smaller than 0.84” and 0.9 respectively, were carried out by use of JMP18 (SAS, Cary, North Carolina, USA) or R (R Core Team, 2019). To assess the stability of the identified cutoffs, we performed a subsampling stability analysis. In each of 20 iterations, a random subset of 70% of cases was drawn without replacement and the recursive partitioning model was refitted. The resulting distributions of cutoff estimates were examined to evaluate robustness against sampling variability. For figure design Affinity Designer software was used (version 1.8.4.693, Serif (Europe) Ltd, Nottingham, UK). The Pearson Chi² test was used for categorical variables in contingency tables. Dunn’s test was chosen as a post-hoc pairwise comparison method following a Kruskal–Wallis test to account for non-parametric distribution of data. A *p*-value < 0.05 was considered as statistically significant. Figure 1 was created using BioRender.com.

## Results

### Study workflow and general patient characteristics of the STX cohort

The overall study workflow is depicted in Fig. 1a. Briefly, STX-derived samples averaging 1 mm³ were subjected to both histopathological as well as molecular diagnostics. Pathological workup of tissue specimens included staining against IDH1_R132H, H3K27M, its control H3K27me3 and ATRX and is subsumed under the term “Routine diagnostic workup (RDW)” in the following. We collected large-scale DNA methylomes using the Human Methylation EPIC array v1. Samples passing quality check by the R package “RnBeads” were further processed [22]. Calibrated scores were collected using the Heidelberg, Bethesda and EpiDiP brain tumor classifiers. Copy number profiling was performed using the Heidelberg brain tumor classifier. Reference-based tumor deconvolution was conducted additionally by use of MethylCIBERSORT and the LUMP algorithm to estimate the cellular composition [23]. Data obtained from RDW and DNA methylation analysis of small tumor samples contributed to integrative, molecular grading of brain tumors according to the 5th edition of the WHO Classification of Central Nervous System Tumours [20]. Methylation data of unclassifiable samples in the HD classifier (calibrated score < 0.84 and no indicative copy number alterations) was subjected to alternative classifier analysis (Bethesda, EpiDiP). Furthermore, the acquired data was used to analyze the thresholds of DNA concentration and cancer cell proportions of stereotaxic biopsies to permit solid molecular grading.

In total, 237 patients with a radiologically diagnosed brain tumor receiving STX and DNA methylation analysis were included in this study (Fig. 1b). There was a preponderance for male patients (60.3% of the cohort; Supplementary Excel File) with median age at diagnosis of 59 years and 94.9% of tumors accounted for newly diagnosed intracranial masses. Based on RDW, tumors were stratified into glioma (227/237), pineal (1/237), mesenchymal/non-meningothelial (1/237) and embryonal tumors (1/237), respectively. 7/237 specimens remained unclassifiable, reaching an overall diagnostic yield of 97.05%. Among gliomas most tumors displayed signs of malignancy and were assigned to the group “H&E malignant diffuse glioma” (131/227). “H&E diffuse glioma” for samples showing a diffuse infiltration pattern but lacking overt signs of malignancy was diagnosed in 86/227 cases, “H&E circumscribed glioma” was diagnosed in 10 out of 227 cases (Fig. 1b).

### General sample characteristics, DNA concentration analysis and quality check of the STX cohort

Intra-operative diagnostic workup of STX samples was performed by a neuropathologist in 97.5% of cases (Supplementary Excel File). Out of 15 samples in median per patient (range 1–37) one sample was subjected to smear preparation during surgery (median 1, range 0–9). Four samples in median per patient were processed for DNA methylation analysis (range 2–9, Supplementary Fig. 1a). The median DNA concentration extracted from STX samples amounted to 6.26 ng/µl (range 0.4–186; Supplementary Fig. 1b, Supplementary Table 1). Next, as the manufacturer recommends a minimum of 250ng of absolute DNA are for the Human Methylation EPIC array analysis, STX DNA samples were stratified into the groups “not measurable” (3.8%, *n* = 9), “>0 < 250ng” (40.9%, *n* = 97, median DNA concentration 3.02 ng/µl, absolute DNA amount on beadchip 135.9 ng) and “>250ng” (55.3%, *n* = 131, absolute DNA amount on beadchip 250 ng; Supplementary Table 1, Supplementary Fig. 1c). The methylation data quality check rendered overall high quality in 232/237 samples (97.9%). 5/237 samples (2.1%, located on five different beadchips) failed quality requirements using the *Greedycut* iterative algorithm implemented in *RnBeads* (see Material and Methods; [22]) and were removed collectively from the data set for further molecular analysis. Information about final reported diagnoses, DNA concentration, DNA methylation classes, MGMT promoter methylation status and CNP of these five samples are provided in Supplementary Table 2.

### Results of HD brain tumor classifier analysis in STX samples

**Fig. 2:**
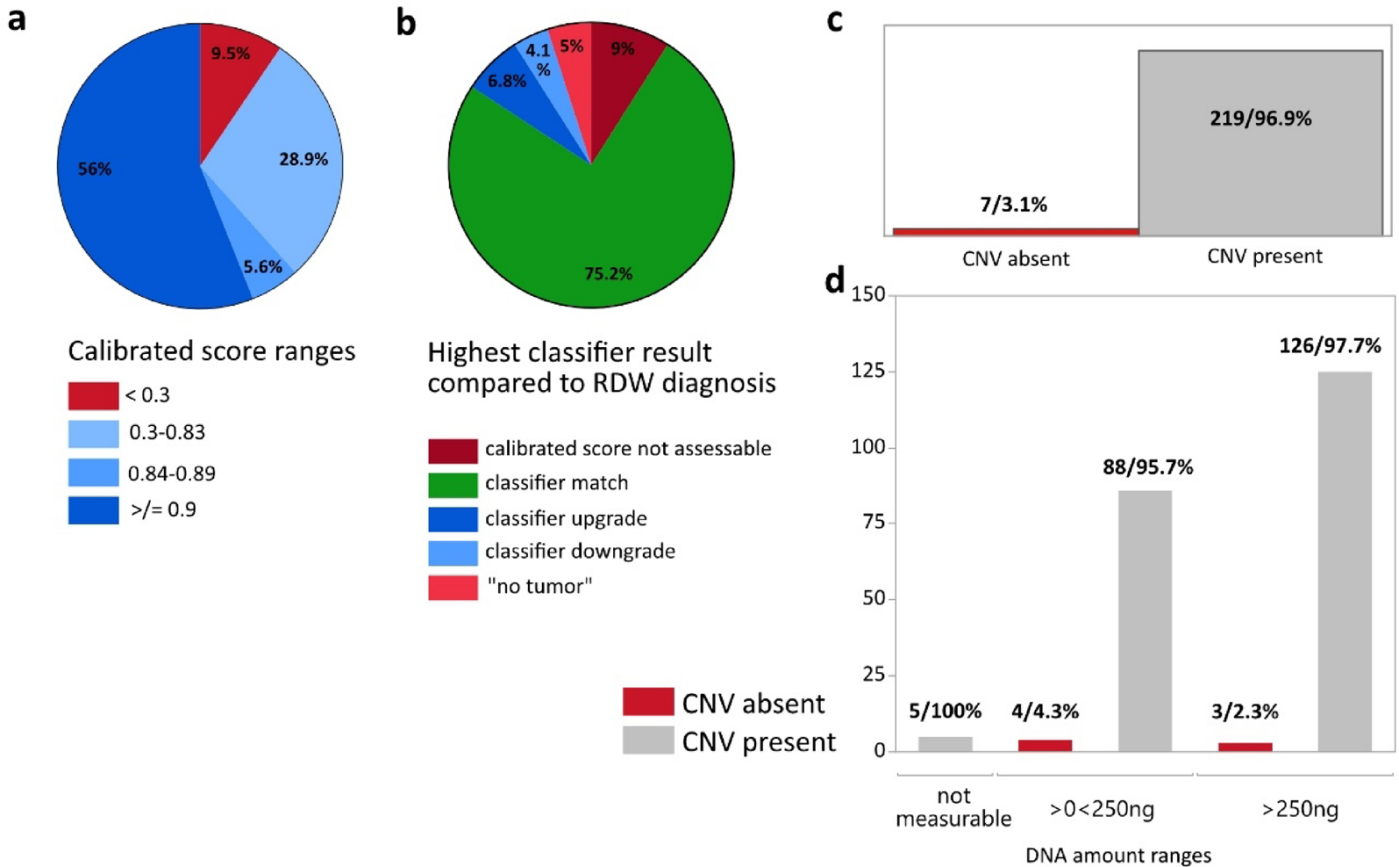
Calibrated scores, copy number profiling and MGMT promoter methylation status in STX samples, HD brain tumor classifier. **a** Frequency of assigned calibrated scores categorized in < 0.3 and ranges 0.3–0.83, 0.84–0.89 and >/=0.9, pie chart. **b** Frequency of assignment of STX samples to categories “not assessable calibrated scores”, “match between calibrated score and histological diagnosis”, “classifier up- or downgrade” and classifier scores suggesting “no tumor” upon the comparison of classifier results with histological diagnoses, pie chart. **c** Absolute and relative numbers of tumors harboring copy number alterations across all brain tumors. **d** Absolute and relative counts of samples showing copy number variations within DNA amount ranges. RDW: routine diagnostic workup

DNA methylation profiles of 90.5% (*n* = 210) of STX samples were assignable to a DNA methylation class by use of the Heidelberg brain tumor classifier v11b4 (total *n* = 232, Fig. 2a). In 56% (*n* = 130) of cases calibrated scores matched with a DNA methylation class (> 0.9). Calibrated scores ranged between 0.84 and 0.9 in 5.6% (*n* = 13) and reached scores between 0.3 and 0.84 in 28.9% (*n* = 67) of cases. HD brain tumor classifier calibrated scores were below 0.3 in 9.5% (*n* = 22) of cases.

Looking closer into diagnostic implications of calibrated scores in combination with RDW diagnoses (*n* = 224 cases in total, removal of H&E unclassified brain lesion and samples not passing QC), highest classifier results mostly suggested assignment to DNA methylation classes which were in line with histology (75%, *n* = 168; Fig. 2b). The calibrated scores did not yield any diagnostic indication in 14.4% (*n* = 32) of cases (“calibrated score not assessable”, 9.4% (*n* = 21), “no tumor”, 5% (*n* = 11)). Classifier results and RDW diagnoses bared discrepancies in 10.7% (*n* = 24) of STX samples: in 4% (*n* = 9) of cases assigned DNA methylation classes implicated lower malignancy than histology, in 6.7% (*n* = 15) of cases higher malignancy, respectively.

Using the STP27 Model MGMT promoter methylation status was determinable in 91.9% of cases and methylated in 50.2% considering glioma only (*n* = 223, exclusion of samples not passing the RnBeads quality check, unclassified brain lesions and non-gliomas; Supplementary Table 3; [3]). The assignment of MGMT promoter methylation status depended on the amount of input DNA (p 0.0008, Pearson Chi² test; Supplementary Fig. 1d).

HD brain tumor calibrated scores significantly depended on DNA amounts too and increased with higher DNA input (Supplementary Fig. 1e, Supplementary Table 4, exclusion of unclassified brain lesions and “no match” calibrated score results). Calibrated scores differed significantly between DNA amount ranges > 0 < 250ng and > 250ng, but > 0 < 250ng samples nevertheless yielded a median calibrated score of 0.91.

After exclusion of unclassified brain lesions, calibrated scores below 0.3 were found in 3 (60%), 9 (9.78%) and 11 (8.53%) cases of DNA amount ranges “not measurable”, “>0 < 250ng” and “>250ng”, respectively, showing that non-measurable DNA concentrations often lead to non-indicative results (Table 1). Under the caveat of differing group sizes, most cases however belonged to the DNA amount range > 250ng (*n* = 11, 47.83%) within the group of calibrated scores below 0.3 (*n* = 23). Importantly, DNA amounts meeting the assay optimum reduced the percentage of allocation to the classifier result “no tumor” (20%, 7.61% and 2.33% for DNA amount ranges “not measurable”, “>0 < 250ng” and “>250ng”, respectively). Looking closer into classifier results however, similar percentages and median calibrated scores of cases eligible for a classifier upgrade, downgrade or allocation to “no tumor” were seen for DNA input samples “>0 < 250ng” and “>250ng” (Table 1, Supplementary Fig. 1f and Supplementary Table 5). Furthermore, median calibrated scores for methylation classes matching with RDW diagnoses did not differ between both DNA amount ranges.

**Table 1:** Contingency table of DNA amount ranges “not measurable”, “>0 < 250ng” and “>250ng” and classifier results in relation to H&E diagnosis

	CS < 0.3	Classifier match compared to H&E diagnosis	Classifier upgrade compared to H&E diagnosis	Classifier downgrade compared to H&E diagnosis	“No tumor“	*n*
*DNA amount range*						
Not measurable	3/60%	1/20%	0/%	0/%	1/20%	5
> 0 < 250ng	9/9.78%	67/72.83%	5/5.43%	4/4.35%	7/7.61%	92
> 250ng	11/8.53%	100/77.52%	10/7.75%	5/3.88%	3/2.33%	129
Σ	23	168	15	9	11	226
*p*	*0.0037*					

*p* value Pearson Chi² test. Calibrated score (CS).

### Copy number profiling in STX samples

In total, 96.9% of STX samples readily showed copy number variations in DNA methylation-based copy number profiling (*n* = 226, Fig. 2c, exclusion of unclassified brain lesions). 0.8% of H&E malignant diffuse gliomas, 4% of H&E diffuse gliomas, 20% of H&E circumscribed gliomas and the embryonal tumor bared no copy number variations, adding up to 3.1% of absent copy number variations overall (Supplementary Fig. 1g). Copy number variations were present in the pineal tumor and hemangioblastoma. Regarding DNA concentration ranges, copy number variations were present in 100% of samples belonging to the group “not measurable”, 95.7% in “>0 < 250ng” and 97.7% of “>250ng” samples, respectively (Fig. 2d).

### Integrative diagnostics for STX samples

**Fig. 3:**
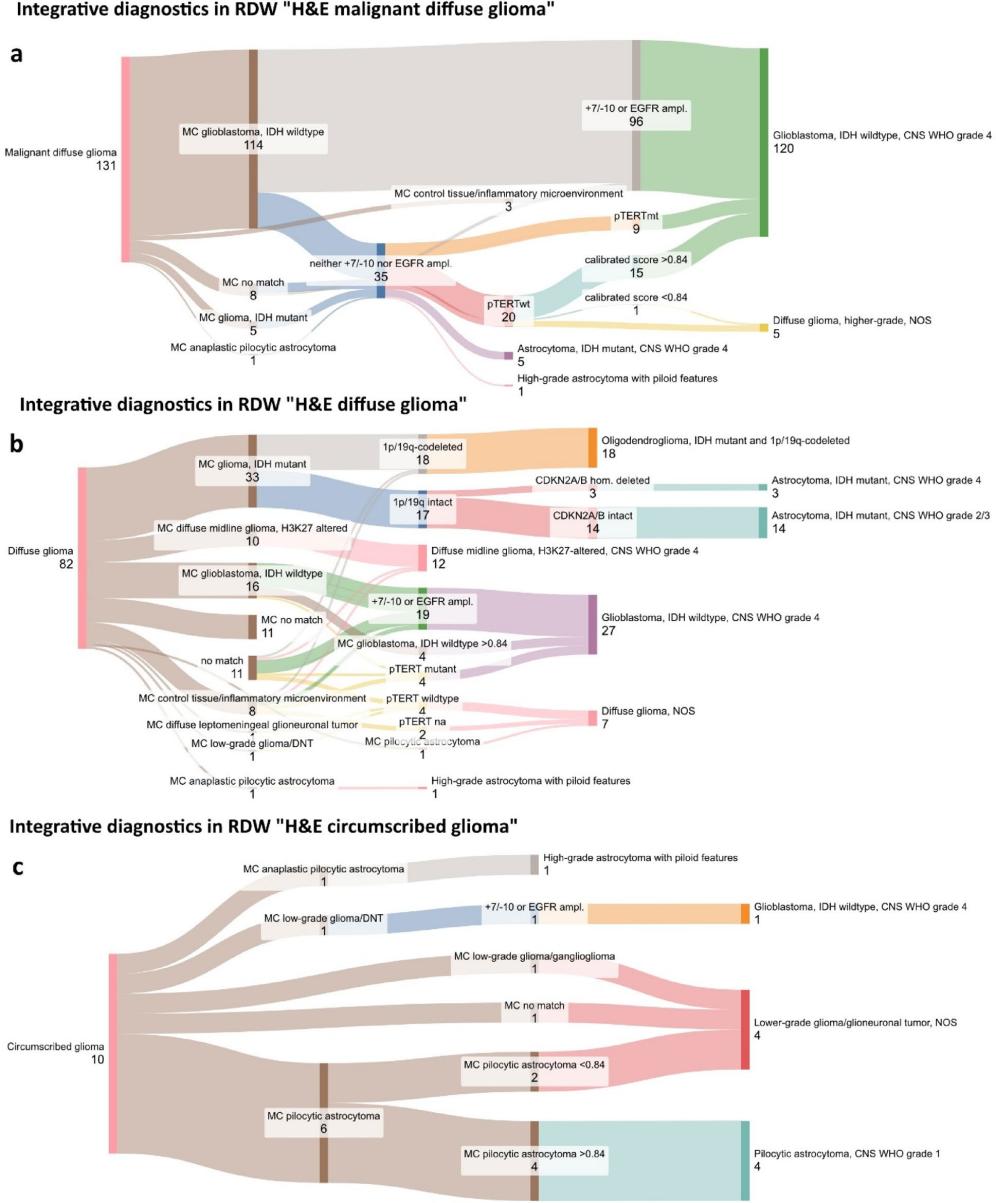
Integrative diagnostics in STX glioma samples using the HD brain tumor classifier.** a** Synopsis of H&E diagnoses, copy number variations, classifier results and *TERT* promoter mutation status (in diffuse gliomas) for integrative diagnostics and molecular grading in routine diagnostic workup “RDW” **a** H&E malignant diffuse gliomas, **b** H&E diffuse gliomas and **c** H&E circumscribed gliomas

Out of 131 STX gliomas with histological signs of malignancy, 114 cases (87%) were allocatable to the methylation class “glioblastoma IDH wildtype” (Fig. 3a). 73.3% (*n* = 96) showed either an *EGFR* amplification or a gain of chromosome 7/loss of chromosome 10. Nine samples harboring no CNV indicative of glioblastoma, IDH wildtype displayed a *TERT* promoter mutation. All samples which were allocated to control tissue methylation classes revealed indicative CNVs (*n* = 3) as did 25% (*n* = 2) of no match-methylation class samples. 120 (91.6%) samples from the H&E malignant diffuse glioma cohort were classified as glioblastoma, IDH wildtype, CNS WHO grade 4. In five (3.8%) samples indicative molecular features were missing, and the samples were assigned to the descriptive diagnosis “Diffuse glioma, higher-grade, NOS” based on histology.

Among 82 diffuse gliomas without evidence of malignancy in RDW, 33 tumors were allocated to the IDH mutant glioma methylation class (Fig. 3b). 18 (22%) showed combined deletion of 1p/19q and were diagnosed as oligodendroglioma, IDH mutant and 1p/19q codeleted and 17 (20.7%) as 1p/19q-intact astrocytoma, IDH mutant including three CNS WHO grade 4 tumors due to a homozygous *CDKN2A/B* deletion. 19 (23.2%) samples showed either an amplification of *EGFR* or a + 7/−10 chromosomal signature indicative of glioblastoma, IDH wildtype, four (4.9%) cases matched HD brain tumor classifier scores for glioblastoma, IDH wildtype and four (4.9%) samples were *TERT* promoter mutant summing up to 27 (32.9%) of glioblastomas, IDH wildtype, CNS WHO grade 4 within the group of H&E diffuse gliomas. Twelve (14.6%) samples were assigned to the diagnosis of diffuse midline glioma, H3K27-altered, CNS WHO grade 4 either by methylation profiling (10/12) or immunohistochemical staining (2/12). The descriptive diagnosis of “Diffuse glioma, NOS” was given to seven (8.5%) samples due to no further indicatory molecular clues.

Most circumscribed gliomas were allotted to the methylation class of pilocytic astrocytoma (60%, *n* = 6, Fig. 3c). One tumor showed CNVs indicative of glioblastoma, IDH wildtype. One tumor was diagnosed as high-grade astrocytoma with piloid features as it scored for the methylation class “anaplastic pilocytic astrocytoma” and showed a homozygous deletion of *CDKN2A/B* (data not shown). Because of no indicative results in methylation class assignment or copy number profiling four cases (40%) were reluctantly classified as “Lower-grade glioma/glioneuronal tumor, NOS”.

The projection of all STX samples into a dimension-reducing space through tSNE analysis showed no clustering of STX methylomes according to input DNA amounts but according to DNA methylation classes (Supplementary Fig. 1h).

50% (*n* = 3) of cases without an indicative histology readily showed CNVs suggestive for a glioblastoma, IDH wildtype, CNS WHO grade 4, 50% (*n* = 3) did not display molecular characteristics relevant for grading (Supplementary Fig. 2a).

Alternative classifier interrogation for analysis of Heidelberg classifier NOS samples and comparison of molecular subtyping of glioblastoma, IDH wildtype.

**Fig. 4:**
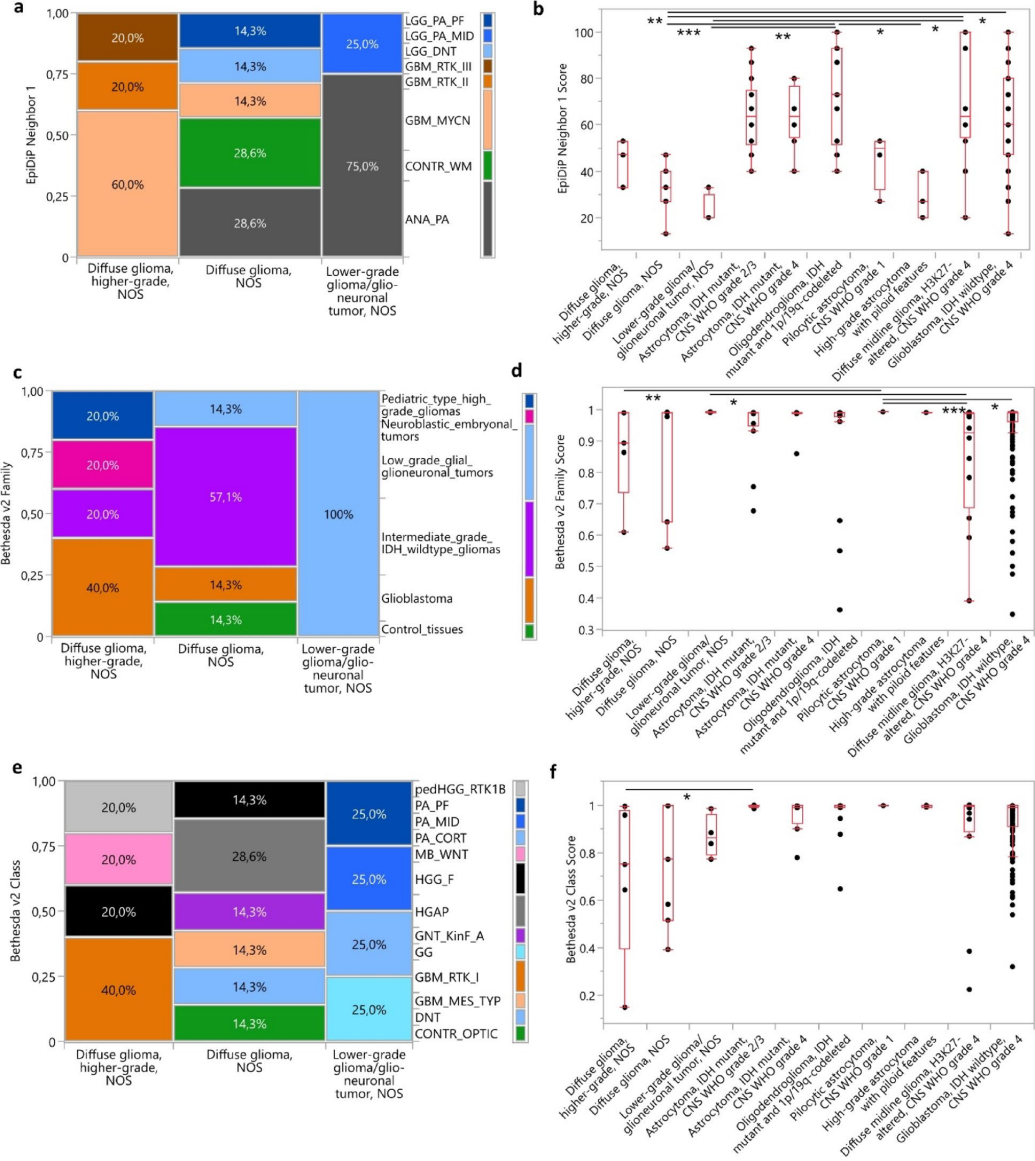
Interrogation of the alternative brain tumor classifiers EpiDiP and Bethesda for samples with failed classification using the HD brain tumor classifier “Diffuse glioma, higher-grade, NOS”, “Diffuse glioma, NOS” and “Lower-grade glioma/glioneuronal tumor, NOS”. **a** Contingency table displaying EpiDiP Neighbor 1 results. **b** Boxplot comparing EpiDiP Neighbor 1 scores of NOS samples to classifiable STX samples. **c** Contingency table displaying Bethesda v2 Family results. **d** Boxplot comparing Bethesda v2 Family scores of NOS samples to classifiable STX samples. **e** Contingency table displaying Bethesda v2 Class results. **f** Boxplot comparing Bethesda v2 Class scores of NOS samples to classifiable STX samples

Samples not classifiable by use of the HD brain tumor classifier (“Diffuse glioma, higher-grade, NOS”, *n* = 5; “Diffuse glioma, NOS”, *n* = 7; “Lower-grade glioma/glioneuronal tumor, NOS”, *n* = 4) were subjected to analyses with the classifiers “EpiDiP” and “Bethesda” (Fig. 4). The highest neighbor score 1 was noted for EpiDiP, for Bethesda results “Family” and “Class” scores were evaluated. Each sample from the “Diffuse glioma, higher-grade, NOS” group neighbored samples from glioblastoma, IDH wildtype groups of different subclasses in EpiDiP (“GBM_MYCN”, “GBM_RTK_II”, “GBM_RTK_III”; Fig. 4a). The neighbor percentage scores did not differ significantly between higher-grade glioma, NOS samples and those unequivocally classified as glioblastoma, IDH wildtype (Fig. 4b). The neighbors of samples from the group of “Diffuse glioma, NOS” were distributed more heterogeneously. While 28.6% neighbored non-neoplastic control tissue methylomes in EpiDiP (“CONTR_WM”, *n* = 2), 42.9% of cases displayed highest scores for gliomas of higher malignancy grade (14.3% “GBM_MYCN”, *n* = 1; 28.6% “Anaplastic astrocytoma with piloid features (ANA_PA)”, *n* = 2). 14.3% were assigned to a neighbor from the lower-grade glioma spectrum (“Low-grade glioma, dysembryoplastic neuroepithelial tumor (LGG_DNT)”, *n* = 1; “Low-grade glioma, pilocytic astrocytoma, posterior fossa (LGG_PA_PF)”, *n* = 1). The level of EpiDiP scores of samples from the “Diffuse glioma, NOS” group was significantly lower than the ones of multiple groups of clearly classified tumors. Three samples (75%) from the “Lower-grade glioma, glioneuronal tumor, NOS” group neighbored the “ANA_PA” methylation group, one case (25%) the methylation group of “Low-grade glioma, pilocytic astrocytoma, midline (LGG_PA_MID)”. The neighbor scores for these NOS tumors were significantly lower when compared to scores of classifiable gliomas. Overall STX samples classified as “oligodendroglioma, IDH mutant and 1p/19q-codeleted” obtained the highest neighbor 1 scores in the EpiDiP classifier (Fig. 4b). The levels of EpiDiP scores depended significantly on input DNA amounts (Supplementary Fig. 2b). Samples with unmeasurable DNA concentrations showed lowest EpiDiP neighbor 1 scores, but no significant difference was remarked between the groups “>0 < 250ng” and “>250ng”. However, samples with DNA input of > 250ng showed the significantly lowest number of neighbors overall (Supplementary Fig. 2c). The lowest number of neighbors was seen for IDH mutant glioma STX samples (Supplementary Fig. 2d).

“Diffuse glioma, higher-grade, NOS” samples were allocated to the Bethesda families “Glioblastoma” (40%, *n* = 2), “Intermediate grade, IDH wildtype glioma” (20%, *n* = 1), “Neuroblastic embryonal tumor” (20%, *n* = 1) and “Pediatric type, high-grade glioma” (20%, *n* = 1; Fig. 4c). Among the group of “Diffuse glioma, NOS” most samples obtained highest scores for “Intermediate grade, IDH wildtype glioma” (57.1, *n* = 4), while one sample (14.3%) was allocated to “Glioblastoma”, “Control tissues” and “Low grade glial, glioneuronal tumor” respectively. The latter family was assigned to all samples from the “Lower-grade glioma, glioneuronal tumor, NOS” group too. The family scores for cases belonging to “Lower-grade glioma, glioneuronal tumor, NOS” were significantly higher than those obtained for STX “Diffuse midline glioma, H3K27-altered” samples (Fig. 4d). An increasing DNA input raised the Bethesda family score significantly (Supplementary Fig. 2e).

Samples from the “Diffuse glioma, higher-grade, NOS” group received Bethesda class allocation to “Glioblastoma, RTK I (GBM_RTK_I)” (40%, n = 2), “High-grade glioma, subtype F (HGG_F)” (20”, *n* = 1), “Medulloblastoma, WNT-activated (MB_WNT)” (20%, *n* = 1) and “Pediatric type high-grade glioma, RTK 1B (pedHGG_RTK1B)” (20%, *n* = 1; Fig. 4e). Bethesda classes assigned to “Diffuse glioma, NOS” samples were “High-grade astrocytoma with piloid features (HGAP)” (28.6%, *n* = 2), “HGG_F” (14.3%, *n* = 1), “(GNT_KinF_A)” (14.3%, *n* = 1), “Glioblastoma, IDH wildtype, mesenchymal, typical (GBM_MES_TYP)” (14.3%, *n* = 1), “DNT” (14.3%, *n* = 1) and “Control tissue (CONTR_OPTIC)” (14.3%, *n* = 1). 75% of samples from the “Lower-grade glioma, glioneuronal tumor, NOS” group were allocatable to pilocytic astrocytoma methylation classes (25%, *n* = 1 each for posterior fossa, midline and cortical) one sample (25%) was assigned to “Ganglioglioma (GG)”. The “Diffuse glioma, higher-grade, NOS” and “Diffuse glioma, NOS” class scores showed a broad range of distribution (Fig. 4f). No significant differences were seen between groups of different amounts of DNA input (Supplementary Fig. 2f).

The most common HD brain tumor classifier glioblastoma, IDH wildtype methylation subclasses “mesenchymal”, “RTK I” and “RTK II” were reproducible using the EpiDiP and Bethesda classifiers (Supplementary Fig. 3a–c). Samples assigned to the HD methylation subclass “midline” and “MYCN” however, differed by use of the Bethesda classifier. They were allocated to Bethesda families “Pediatric type, high-grade glioma” (midline: 75%, *n* = 3, MYCN: 100%, *n* = 1) and “Intermediate grade, IDH wildtype glioma” (midline: 25%, *n* = 1) as well as to Bethesda classes “HGG_B” (midline: 25%, *n* = 1), pediatric type high-grade gliomas (midline: pedHGG_RTK1A 50%, *n* = 2; pedHGG_RTK1C 25%, *n* = 1) and ped_HGG_A (MYCN, 100%, *n* = 1).

### Tumor deconvolution and recursive partition modeling of minimum input threshold in STX samples

**Fig. 5:**
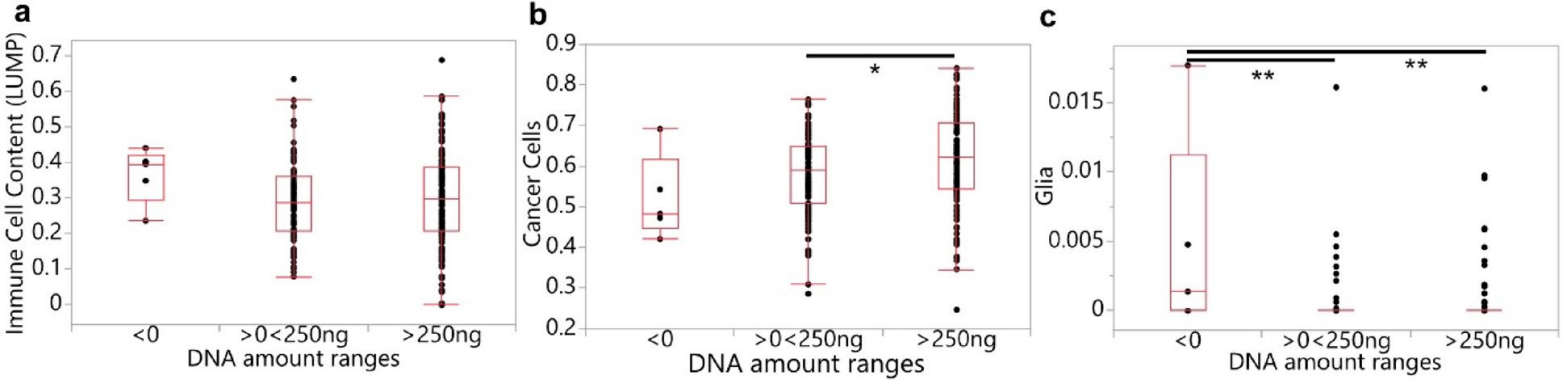
Tumor deconvolution in STX samples. Reference-based tumor deconvolution, grouped by input DNA amount into “<0”, “>0 < 250ng” and “>250ng” **a** Immune cell content computation, LUMP algorithm, **b** cancer cell proportions, MethylCIBERSORT, **c** glial proportions, MethylCIBERSORT. * *p* < 0.05, ** *p* < 0.001

For tumor purity estimation all STX samples meeting QC criteria and showing tumor tissue in H&E staining (*n* = 226) were subjected to in silico reference-based tumor deconvolution. LUMP estimates of overall leukocyte ratios did not vary between different groups of DNA input (Fig. 5a). Cancer cell proportions were significantly higher in samples with a DNA input amount bigger than 250ng than in those with measurable but lower DNA amount than 250ng, while the proportions of glial cells were highest in the sample group without measurable DNA concentration (Fig. 5b, c).

Recursive partition modeling pointed towards differing and brain tumor classifier dependent minimum analyte inputs to reach valuable calibrated scores. By choosing a cut-off of 1.21 ng/µl for input DNA concentration the probability of allocation to calibrated scores bigger than 0.84 increased from 0.31 to 0.71 using the Heidelberg brain tumor classifier and a cancer cell threshold bigger than 53% was needed to raise the probability of robust allocation from 0.43 to 0.76 (Table 2a). DNA concentrations and cancer cell proportions amounted to 3.18 ng/µl and 62%, respectively, when a Heidelberg brain tumor classifier score of 0.9 was aimed for (Table 2b). A Bethesda Family Score over 0.9 was reached when a DNA amount bigger than 2.95 ng/µl was used as input and/or the sample contained a cancer cell proportion bigger than 37.4%. Meeting these criteria the allocation probability increased from 0.69 to 0.88 for DNA concentration and 0.47 to 0.85 for cancer cell proportion, respectively (Table 2c). The alignment with a Bethesda Class Score bigger than 0.9 improved from 0.57 to 0.8 when DNA concentration was higher than 1.59 ng/µl and from 0.77 to 0.97 when the cancer cell proportion was as high as 79.4% (Table 2d). While significant split *p* values of *p* 0.000999 and *p* 0.000398 were reached when choosing the cancer cell proportion cut-offs 53% and 62% to dichotomize the samples for the allocation to Heidelberg classifier scores > 0.84 and > 0.9 respectively, these cut-offs explained only a small proportion of the data variance as indicated by low R². Significant split *p* values or strong R² were not reached for the other recursive partition models. Consistently, upon resampling-based validation by repeated random subsampling the cancer cell proportion showed high LogWorth values (defined as − log₁₀(p), where higher values indicate greater statistical significance) for stratification of the samples to high Heidelberg classifier scores, but this association was not linear (Supplementary Fig. 4 and Supplementary Table 6). Using DNA concentrations as predictors of robust classifier allocation was not feasible, nor was dichotomization according to DNA concentration or cancer cell content for Bethesda classification.

**Table 2:** Recursive partitioning for DNA concentration (ng/µl) and cancer cell proportion (%) needed to reach a (a) methylation class classifier score (CS) bigger than 0.84 in the Heidelberg brain tumor classifier, (b) methylation class classifier score (CS) bigger than 0.9 in the Heidelberg brain tumor classifier, (c) family classifier score (CS) bigger than 0.9 in the Bethesda brain tumor classifier, (d) class classifier score (CS) bigger than 0.9 in the Bethesda brain tumor classifier

	DNA concentration (ng/µl) > / = 1.21	DNA concentration (ng/µl) < 1.21
*(a) Heidelberg brain tumor classifier, methylation class, calibrated score cut-off 0.84*		
n	192	14
G^2	230	16.75
CS < 0.84 probability	0.29	0.69
CS > 0.84 probability	0.71	0.31
R^2^	0.039	
Split *p* value	0.1037	

	Cancer cells (%) > / = 53	Cancer cells (%) < 53
n	157	49
G^2	171.46	66.92
CS < 0.84 probability	0.24	0.57
CS > 0.84 probability	0.76	0.43
R^2^	0.072	
Split *p* value	*0.000999*	

	DNA concentration (ng/µl) > / = 3.18	DNA concentration (ng/µl) < 3.18
*(b) Heidelberg brain tumor classifier, methylation class, calibrated score cut-off 0.9*		
n	163	43
G^2	205.61	59.03
CS < 0.9 probability	0.33	0.55
CS > 0.9 probability	0.67	0.45
R^2^	0.028	
Split *p* value	0.3152	

	Cancer cells (%) > / = 62	Cancer cells (%) < 62
n	94	112
G^2	97.31	155.23
CS < 0.9 probability	0.21	0.51
CS > 0.9 probability	0.79	0.49
R^2^	0.073	
Split *p* value	*0.000398*	

	DNA concentration (ng/µl) > / = 2.95	DNA concentration (ng/µl) < 2.95
*(c) Bethesda brain tumor classifier, family*		
n	178	48
G^2	133.15	59.62
CS < 0.9 probability	0.12	0.31
CS > 0.9 probability	0.88	0.69
R^2^	0.043	
Split *p* value	0.2409	

	Cancer cells (%) > / = 37.4	Cancer cells (%) < 37.4
n	221	5
G^2	189.76	6.73
CS < 0.9 probability	0.15	0.53
CS > 0.9 probability	0.85	0.47
R^2^	0.024	
Split *p* value	0.8743	

	DNA concentration (ng/µl) > / = 1.59	DNA concentration (ng/µl) < 1.59
*(d) Bethesda brain tumor classifier, class*		
n	203	23
G^2	201.49	31.49
CS < 0.9 probability	0.2	0.43
CS > 0.9 probability	0.8	0.57
R^2^	0.025	
Split *p* value	0.6385	

	Cancer cells (%) > / = 79.5	Cancer cells (%) < 79.5
n	7	219
G^2	0	235.31
CS < 0.9 probability	0.028	0.23
CS > 0.9 probability	0.97	0.77
R^2^	0.013	
Split *p* value	0.975	

## Discussion

Contemporary molecular pathology moves towards increasingly precise and multifaceted analysis techniques, while simultaneously decreasing tissue sample sizes needed as an input [8, 34]. In case of tumors with an unfavorable location like CNS midline structures or a vast diffusely infiltrative growth pattern, the technical developments permitting the use of low sample amounts are promising. Specifications about DNA contents of STX-derived biopsies and their suitability for clinical-grade DNA methylation profiling, however, remain unknown so far.

Previous studies on STX biopsies questioning the accuracy and eligibility for histopathological and molecular analysis do either not specify on sample sizes or numbers, or provide vague information about the methodological repertoire used on those samples of limited size [11, 19, 21, 26, 29, 35]. Hamisch et al. for instance reported on the analysis of “two to eight tissue samples of 3 to 10 mm” from 511 STX-biopsied deep-seated or midline CNS lesions. The authors stated to meet WHO 2016 conform diagnostic standards in all tumour patients, but do not enumerate used diagnostic approaches [12]. Jain et al. who on one hand specified on sample numbers taken by use of a 2-mm Leksell–Sedan punch biopsy forceps and showed that a higher histology-based diagnostic accuracy of 88.2% vs. 76.5% was achieved when three compared to one diagnostic specimen were obtained, do on the other hand not provide proportions of tumor cells as a reference point [16]. Albeit not performed on STX samples from brain tumors, but artificially reduced DNA input samples of a different cancer entity, it was shown, that 50ng low input samples still produced CpG probe detection rates of up to 94.96% but with the caveat of a high dispersion of copy number profiles [37]. Since brain tumor classification typically benefits from both high classifier scores and sharp copy number profiles, defining a standard for DNA input becomes particularly complex.

With this study on 237 STX brain tumor samples of minimal size, we were able to determine a minimum input DNA concentration of 1.21 ng/µl (equaling an absolute amount of 54.5 ng input DNA) to reach a robust tumor classification > 0.84 using the EPIC array and the Heidelberg brain tumor classifier. Samples harboring a DNA concentration of 1.21 ng/µl were likely to match with a DNA methylation class with a probability of 0.71. However, with this cut-off explaining only a limited proportion of data variation and originating from a single-center retrospective STX cohort it should be regarded as exploratory. In line with this, lower DNA concentrations did not necessarily preclude successful allocation to a DNA methylation class. Further testing on external cohorts would be mandatory to explore the potential generalizability of this cut-off. With a high diagnostic yield, measurable DNA content in 96.2% of specimen, fulfillment of QC criteria in 97.9%, detectable CNVs in 96.9% and no clustering according to lower DNA input in tSNE analysis samples derived from STX overall qualified well for neuropathological diagnostics. Furthermore, using four samples of 1 mm³ of size in median, STX samples were suitable for state-of-the-art brain tumor classification since they allotted to HD classifier methylation classes in 90.5% and to a classifier score result bigger than 0.84 in 61.6% [6].

5/237 samples (2.1%) did not pass the quality control criteria in the Greedycut/RnBeads pipeline, which rely on on-chip control CpG probes. For two samples (“bad QC number 4 and 5”) this may be related to very low amounts of tumor DNA, because neither the DNA concentration nor the MGMT promoter methylation status and copy number alterations were assessable. The detection of IDH1_R132H-positive tumor cells in “bad QC sample 5” however allowed for a final diagnosis of IDH mutant glioma. “Bad QC samples 1–3” on the other hand rather harbored deleterious overall sample quality since “bad QC sample 1”, which showed IDH1_R132H-immunoreactive tumor cells, received scores beneath the cut-off of 0.9 for the methylation class of IDH-mutant glioma and displayed copy number alterations, “bad QC sample 2” showed an adequate DNA concentration of 7.3 ng/µl and copy number alterations were readily detectable in “bad QC sample 3”.

Under the caveat of missing validation of the STP27 model, conclusive MGMT promoter methylation status was evaluable in 91.9% of cases. Although higher DNA amounts were associated with less frequent allocation to non-neoplastic methylation classes as well as higher classifier scores in median in general, solid scores bigger than 0.9 indicative of matches to methylation classes were readily reached within the input DNA group “>0 < 250ng”. Along that line, we found CNVs in all samples with non-measurable DNA content while most unclassifiable samples (calibrated scores below 0.3) belonged to the DNA input group “>250ng” instead of the ones with lower DNA amount outlining sample quality as important factor for successful classification. This STX sample series corroborates findings by Lara-Almunia et al., who argue for DNA concentration, ability of copy number profiling and cancer cell content as being superior determinants of diagnosis quality and state-of-the-art molecular grading to mere sample numbers [19]. As the manufacturer suggests a total DNA amount of 250ng per sample equaling a concentration of 5.56 ng/µl, our findings point towards a substantially lower need of input while paving the way for DNA methylation-based molecular analysis for small CNS tumor samples. However, it is important to highlight that our cohort of stereotaxic brain tumor samples mainly incorporated diffuse gliomas. Consequently, the results need to be interpreted cautiously since input DNA concentration metrics might vary in brain tumors other than gliomas.

A cancer cell content of 70–80% at a minimum is recommended for array-based DNA methylation analysis which mostly is assessed microscopically [4, 6, 17, 36]. Especially tumor samples collected by stereotaxic biopsy are prone to not reaching this proposed landmark. Using the LUMP algorithm STX specimen with a non-measurable or DNA content below 250ng did not show higher leukocyte infiltration than samples meeting the assay input optimum. The reference-based tumor deconvolution with MethylCIBERSORT however pointed towards highest glia cell ratios in non-measurable samples and a higher cancer cell content in samples of DNA yielding at least 250ng of DNA compared to a measurable but smaller input than 250ng. These results indicate that overall higher DNA input is most likely accompanied by higher tumor cell density and less resident cells, where the inflammatory microenvironment might play a smaller role as confounder. In absence of indicative classifier results, CNVs paved the way for integrative diagnostics. Among H&E malignant diffuse gliomas, 25% (*n* = 2) of “no match” and 100% (*n* = 3) of control tissue classifier allocation showed chromosomal aberrations pathognomonic for glioblastoma, IDH wildtype; 23.3% (*n* = 19) of H&E diffuse gliomas and 10% (*n* = 1) of H&E circumscribed gliomas lacking malignancy signs in histology as well as 50% (*n* = 3) of histologically unclassifiable brain lesions showed indicative CNVs. These findings argue for the need for alternative pre-analytic quality parameters and do not preclude the usability of small or low input samples, like from tumor infiltration borders, per se from DNA methylation-based analysis [25]. With a cancer cell content of 53% and 62% in the Heidelberg brain tumor classifier, the probability of assignment to a matching calibrated score of at least 0.84 and 0.9 in our exploratory, STX-cohort fitted approach reached 0.81 and 0.79, respectively. Judging from the recursive partition modelling supported by resampling-based validation by repeated random subsampling we conclude that cancer cell proportions can serve as potent predictors of high classifier scores, but the observed associations showed no linearity arguing for a sample-inherent factor, like DNA quality for instance, to drive classification success.

Although reference-based tumor deconvolution approaches might be error-prone due to a dependency on accurate reference data sets, their implementation in routine diagnostics might serve as an important tool for the assessment of sample quality. Indeed, recent studies showed that computational approaches intended at purifying the cancer cell methylation signal by removal of non-cancer cellular methylation signatures facilitated diagnostics [18, 38]. These in silico-methods allowed for an assignability of priorly non-classifiable samples to pre-defined tumor clusters as well as a robust classification using the Heidelberg brain tumor classifier, respectively [18, 38]. The further development of tools to enhance cancer cell-specific methylation signatures will help to state diagnoses more precisely while economizing DNA input and is of utmost interest for samples of limited size, like STX-derived ones.

Most STX samples reached reasonably high calibrated scores enabling integrative diagnoses. In detail 61.6% % of samples displayed a score bigger than 0.84. A match between the classifier result and histology was noted for 75.2% of samples, 10.9% of samples were subjected to a refined diagnosis based on a classifier up- or downgrade of the prior histological diagnosis. Overall, the ratio of classifiable tumor samples derived from STX aligned with results gathered from resection cases [17, 38]. Opposed to that, the methylomes of 9.5% cases did not match with a methylation class leading to 14% of STX samples without indicative result when classifier scores were considered solely. Among STX samples the ratio of unassignable classifier scores was thus higher than reported for resections [9, 17]. While in line with another study observing significantly lower median classifier scores in lower input samples this concerned samples with non-measurable DNA amounts only in our study, since those with a “> 0 < 250 ng” input still produced median scores above 0.84 [38]. Furthermore, additional analysis of copy number profiles as well as *TERT* promoter mutations in selected cases, led to final integrated diagnoses for 96.2% of H&E malignant diffuse gliomas, 91.5% of H&E diffuse gliomas and 60% of H&E circumscribed gliomas using the Heidelberg brain tumor classifier [6].

While methylation-based tumor classification contributes substantially to neuropathological diagnoses some cases stay unresolved. The ratio of unclassifiable STX samples was highest among H&E circumscribed gliomas in our series. Pediatric type gliomas, as well as glioneuronal and lower-grade gliomas also occurring more frequently in children often issue a challenge to neuropathologists. In a cohort of pediatric brain tumors originating from the MNP2.0 trial Sill et al. observed higher-grade tumor histology opposed to lower-grade tumor methylation signatures hampering clinical decisions for treatment [31]. Along that line, using the alternative brain tumor classifier EpiDiP for NOS samples among the H&E circumscribed glioma group, which lacked any signs of higher-grade glioma, 75% (*n* = 3/4) displayed highest similarity with the methylation class of anaplastic astrocytomas with piloid features. The Bethesda classifier on the contrary allotted 100% of these cases (*n* = 4/4) to the methylation family of low-grade glial/glioneuronal tumors unequivocally. The discrepancies between both histology and molecular characteristics as well as classifier results highlight the complexity of lower-grade and circumscribed gliomas and argue for the exhaustion of molecular analyses by incorporation of next-generation sequencing into integrative diagnostics [14].

The interrogation of the alternative brain tumor classifier EpiDiP which calculates percentage scores of methylation signature similarity to neighboring tumors for unresolved cases by the Heidelberg classifier indicated the clearest results for specimen with histological signs of malignancy and optimal DNA input amounts. All “Higher-grade glioma, NOS” samples neighbored glioblastoma, IDH-wildtype representing concordance in terms of biological aggressiveness rating too. Their EpiDiP percentage scores did not differ significantly from the ones of glioblastoma, IDH-wildtype and they displayed the lowest number of candidate neighbors arguing for unequivocal methylation profiling. Classification of NOS samples using the Bethesda classifier rendered a more diverse spectrum of allocation to methylation classes potentially implying a more nuanced estimation of tumor malignancy. While by use of the Bethesda classifier all “Higher-grade glioma, NOS” samples were allocated to cancer methylation families, some received scores for gliomas of intermediate grade or even higher-grade, but non-glioma entities. These differences between classifiers could be explained by differing brain tumor methylation signatures used as reference by the Bethesda classifier, partially ambivalent signatures, rare neoplasms not yet strictly defined on DNA methylation level and in part also by the computational approach for classification [1]. Due to missing signs of malignancy in histology as well as indicative CNVs and HD classifier scores STX “Diffuse glioma, NOS” and “Lower-grade glioma, glioneuronal tumor, NOS” samples were most challenging to classify. Likewise, the spectrum of assigned methylation classes and family scores, respectively, suggested by the alternative brain tumor classifiers EpiDiP and Bethesda varied notably. For “Diffuse glioma, NOS” these allocations encompassed the classes “control tissue”, “Glioblastoma, IDH-wildtype” or also lower-grade glioma, for “Lower-grade glioma, glioneuronal tumor, NOS” allotment to lower-grade gliomas or “higher-grade astrocytoma with piloid features” was computed. Adding to the broad range of tumor subtypes and CNS WHO grades both NOS groups were characterized by rather lower EpiDiP percentage scores. These results argue for a less clear similarity of DNA methylation signatures with reference cases underscoring the difficulties in integrative neuropathological diagnostics. While the limited size of the STX samples might add to this, recent literature rather points at the pre-classifier “NOS status” of some samples to cause problems for final classification, since the proportion of successfully classified NOS specimen in a non-STX cohort was comparable to our STX series [38]. Since higher input DNA amounts reduced the number of groups while elevating the scores of EpiDiP methylation class allocation and Bethesda methylation family scores were significantly higher within the > 250 ng input group, the implementation of in silico methylation signal purification algorithms prior to classification is very likely to further ameliorate the classification robustness in STX samples [18]. The comparison of brain tumor classifier allocation to molecular subtypes within STX glioblastoma, IDH wildtype yielded concordant results for cases without involvement of the midline. Despite their different computational models, the three different classifiers agreed upon the assignment of the most frequent molecular subtypes “mesenchymal”, “RTK 1” and “RTK 2” in most cases which argues for well-defined methylation signatures in most common brain tumors. Glioblastomas located in the midline however caused classifier disagreement. 25% (*n* = 1) of those samples allotted to control tissue methylation classes in the EpiDiP and Bethesda classifiers and 75% (*n* = 3) samples were matched with pediatric type high-grade gliomas by Bethesda. These results corroborate the need for additional diagnostic tools like next generation sequencing and cautious tumor classification for higher grade gliomas of midline location since CNVs and genomic alterations are known to differ from glioblastoma, IDH-wildtype with “classic” location [27].

## Conclusions

With the Heidelberg and Bethesda brain tumor classifiers, two potent and potentially also complimentary tools are at hand for neuropathological diagnostics. The Heidelberg classifier worked robustly in our single center retrospective cohort with a calculated cancer cell content of 53%. Assessment of 1p/19q co-deletion, IDH mutation and MGMT promoter methylation status only does not satisfy current neuropathological standards anymore; additionally, copy number profiling emerges as diagnostically significant factor. Under the caveat of circumscribed and midline gliomas other than histone 3 mutants, STX samples stand up to DNA methylation-based molecular grading and copy number profiling while presenting as full-featured source of diagnostic tissue.

In summary, while our data suggest that STX-derived samples can support DNA methylation-based diagnostics well below current input recommendations, the proposed thresholds are exploratory in nature and reflect the characteristics of a single-center, glioma-dominated cohort. Prospective multi-center validation across a broader spectrum of CNS tumor entities would be essential prior to the implementation of such cut-offs in routine clinical workflows.

## Supplementary Information

Below is the link to the electronic supplementary material.

Supplementary Material 1

Supplementary Material 2

## Data Availability

All data relevant to the study, analyzed and/or generated within this study are included in the article or uploaded as supplementary information. All other data and raw data IDAT files will be made accessible via the Gene Expression Omnibus platform (GEO, https://www.ncbi.nlm.nih.gov/geo/; GSE328441).

## Abbreviations

CDKN2A/B: Cyclin-Dependent Kinase Inhibitor 2 A/B
CNS: Central nervous system
CNVs: Copy number variations
CpG: Cytosine-phosphate-Guanine
CS: Classifier score
EGFR: Epidermal growth factor receptor
FFPE: Formalin-Fixed and Paraffin-Embedded
H3K27M: Histone 3 lysine 27 methionine
IDH: Isocitrate dehydrogenase
LUMP: Leukocytes unmethylated for purity
MGMT: O-6-methylguanine-DNA methyltransferase
NOS: Not otherwise specified
RDW: Routine diagnostic workup
STX: Stereotaxic biopsy
TERT: Telomerase reverse transcriptase
tSNE: t-distributed stochastic neighbor embedding
WHO: World Health Organization
