# Supplementary material for: Methylation profiling and alternative classification approaches in a glioma-enriched FFPE stereotaxic biopsy cohort
Source: Acta Neuropathol Commun. 2026 Jul 22;14:155. doi: 10.1186/s40478-026-02382-z (PMC13393821; doi:10.1186/s40478-026-02382-z)
Supplement: Supplementary file 2 — Supplementary Material 2 [file 40478_2026_2382_MOESM2_ESM.docx]

**Supplementary Figures**


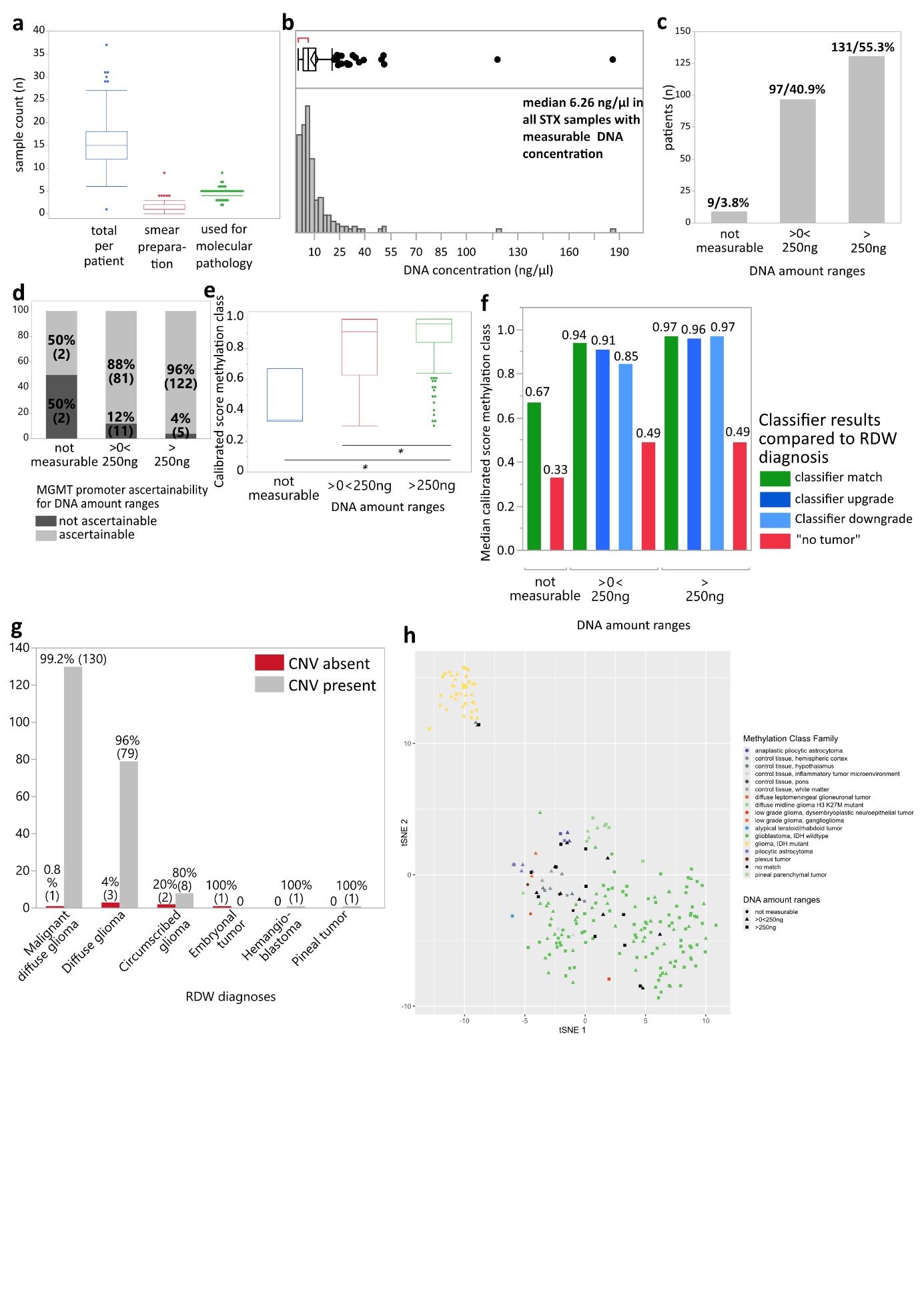


**Supplementary Figure 1. a)** Absolute numbers of STX samples collected per patient, of samples subjected to intra-surgical smear preparation and of samples used for molecular pathological analysis. **b)** Median overall DNA concentration across all STX samples with measurable DNA concentrations. **c)** Allocation of absolute and relative numbers of pooled STX samples per patient to three DNA amount ranges. **d)** Frequency of samples with or without ascertainability of MGMT promoter methylation status with regard to different DNA amount ranges. **e)** Distribution of methylation class calibrated scores reached within DNA amount ranges. ** p<0.05*. **f)** Median methylation class calibrated scores reached for classifier matches/upgrade/downgrade or allocation to “no tumor” compared to histological diagnoses, respectively, in DNA amount ranges. **g)** Percentages of detectable copy number variations (CNV) for every brain tumor entity separately. **h)** Dimension-reducing projection of STX samples in tSNE analysis with regard to methylation class family and DNA amount ranges. RDW: routine diagnostic workup.


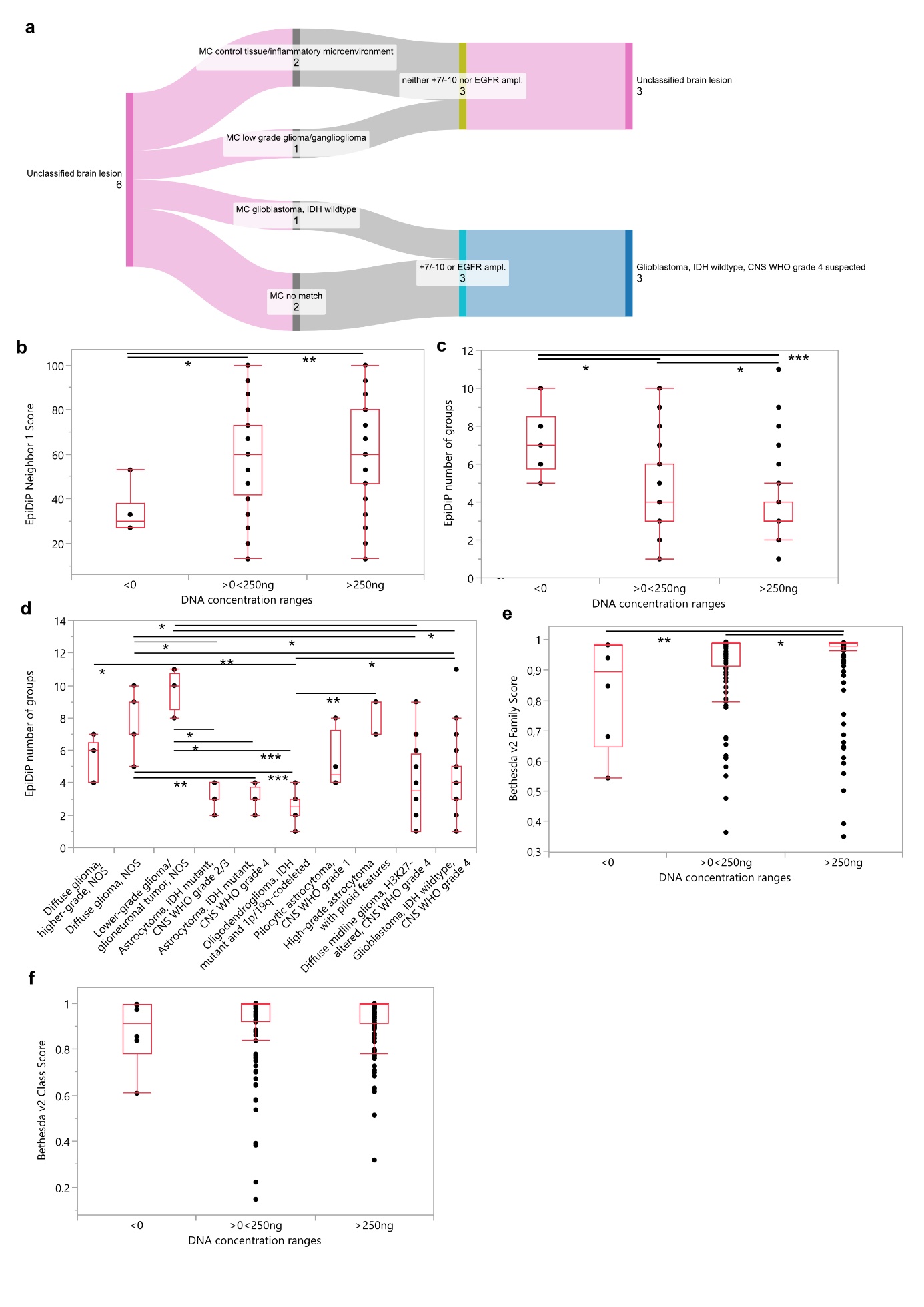


**Supplementary Figure 2: a)** Synopsis of Heidelberg brain tumor classifier results and copy number variations for integrative diagnostics and molecular grading in H&E unclassifiable lesions. **b)** EpiDiP Neighbor 1 results for STX samples stratified according to input DNA amount. **c)** EpiDiP number of groups for STX samples stratified according to input DNA amount. **d)** EpiDiP number of groups for NOS cases compared to classifiable STX samples using the Heidelberg brain tumor classifier. **e)** Bethesda v2 Family Scores for STX samples stratified according to input DNA amount. **f)** Bethesda v2 Class Scores for STX samples stratified according to input DNA amount.


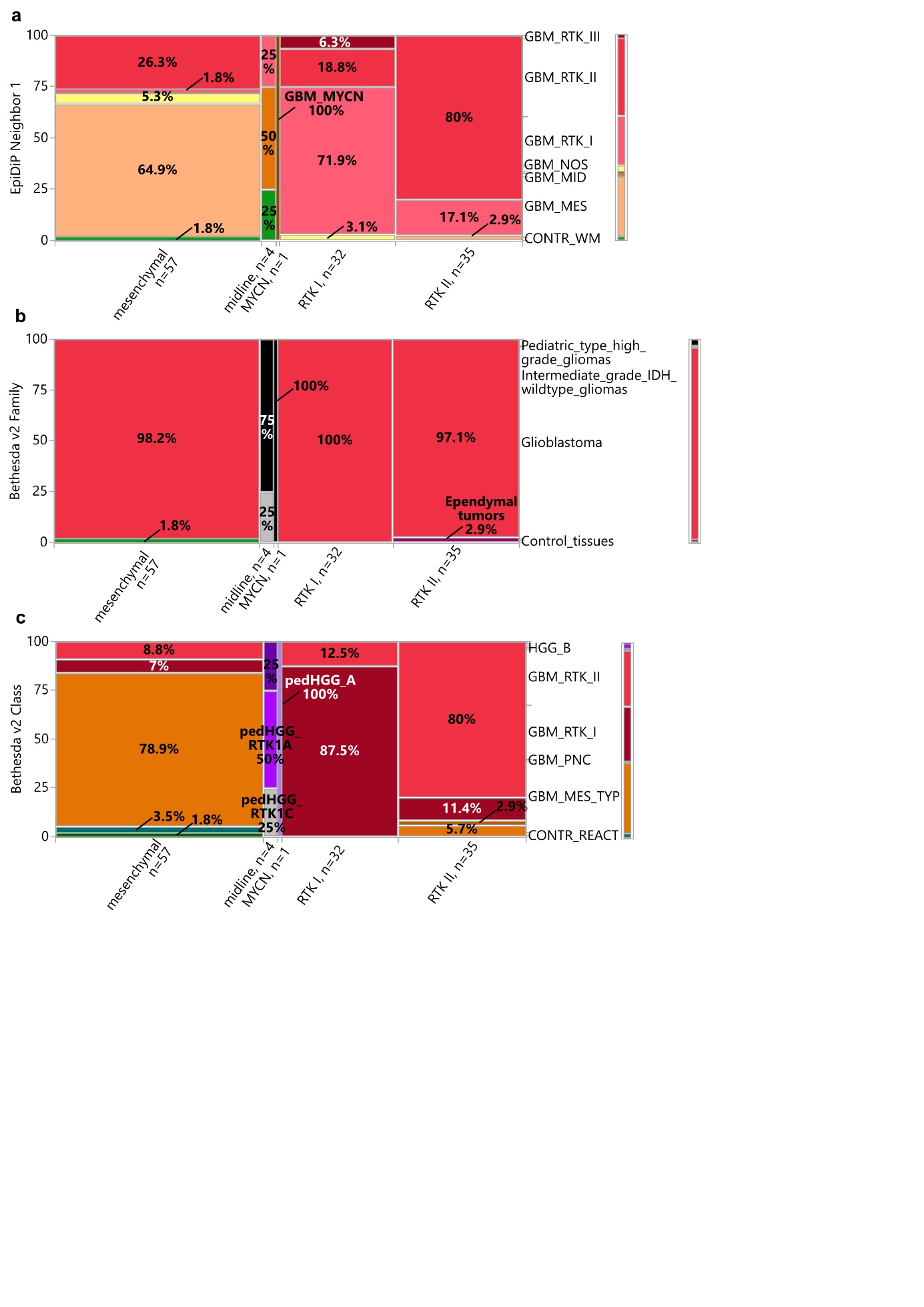


**Supplementary Figure 3:** STX glioblastoma, IDH-wildtype molecular subclass analysis across classifiers in comparison to Heidelberg brain tumor classifier molecular subclasses. **a)** EpiDiP Neighbor 1 results, **b)** Bethesda v2 Family results and **c)** Bethesda v2 Class results.


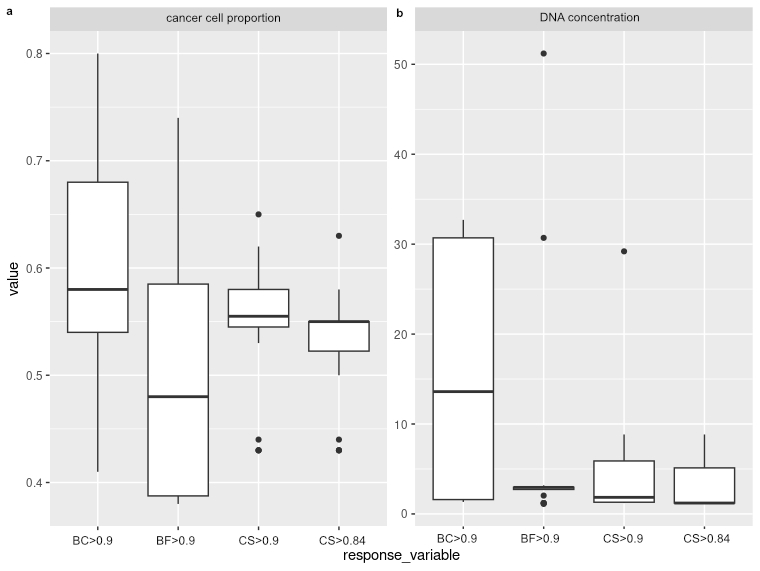


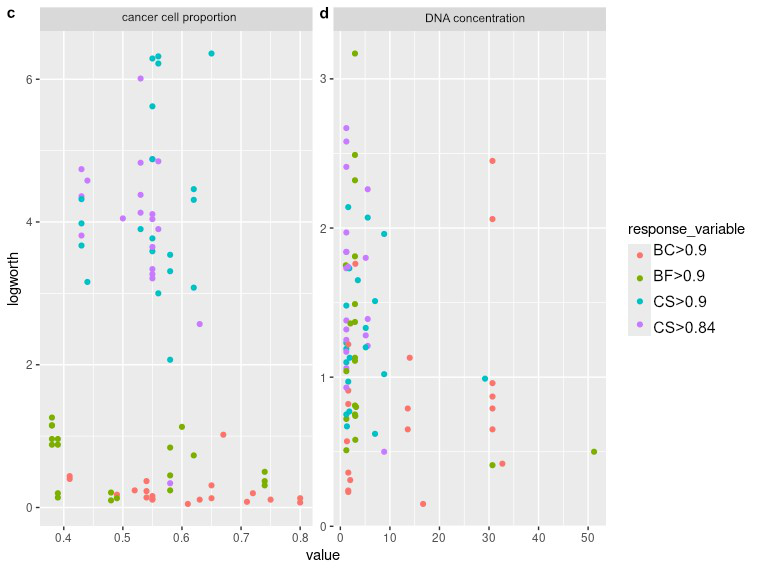


**Supplementary Figure 4:** Subsampling stability analysis, 20 iterations, drawing of a random subset of 70% of cases without replacement for re-fitting of the recursive partitioning model for values for the response variables **a)** “cancer cell proportion” and **b)** “DNA concentration” as well as for LogWorth values for **c)** “cancer cell proportion” and **d)** “DNA concentration”. BC: Bethesda brain tumor classifier, class; BF: Bethesda brain tumor classifier, family; CS: Heidelberg brain tumor classifier, calibrated score.

**Supplementary Tables**

|  | **All samples with measurable DNA concentrations** | **DNA amount >0<250ng** | **DNA amount >250ng** |
| --- | --- | --- | --- |
| **n** | 228 | 97 | 131 |
| **DNA concentration (ng/ul)** |  |  |  |
| **Mean** | 9.99 | 3.03 | 15.15 |
| **Standard deviation** | 16.19 | 1.53 | 19.83 |
| **Mean standard error** | 1.07 | 0.15 | 1.73 |
| **Mean CI lower** | 7.88 | 2.73 | 11.72 |
| **Mean CI upper** | 12.11 | 3.34 | 18.57 |
| **Median** | 6.26 | 3.02 | 9.04 |
| **Min** | 0.4 | 0.4 | 5.56 |
| **Max** | 186 | 5.52 | 186 |
| **25% quartile** | 3.49 | 1.73 | 7.2 |
| **75% quartile** | 10.75 | 4.44 | 15.3 |
| **Absolute DNA amount subjected to analysis for >0<250ng samples** |  |  |  |
| **Mean** |  | 136.56 |  |
| **Standard deviation** |  | 68.64 |  |
| **Mean standard error** |  | 6.97 |  |
| **Mean CI lower** |  | 122.73 |  |
| **Mean CI upper** |  | 150.38 |  |
| **Median** |  | 135.9 |  |
| **Min** |  | 18 |  |
| **Max** |  | 248.4 |  |
| **25% quartile** |  | 77.85 |  |
| **75% quartile** |  | 199.8 |  |

**Supplementary Table 1.** General statistical and quartile values of overall DNA concentrations and absolute DNA amount within DNA amount ranges >0<250ng and >250ng.

| **Bad QC sample ID** | **Final integrative diagnosis** | **DNA concentration (ng/µl)** | **Methylation class** | **Calibrated Score Methylation Class** | **MGMT promoter** | **CNV** |
| --- | --- | --- | --- | --- | --- | --- |
| 1 | Glioma, IDH mutant | 0.4 | Glioma, IDH mutant | 0.39 | n.a. | Present |
| 2 | Glioma, IDH1_R132H negative | 7.3 | Plexus tumor | 0.47 | Methylated | Absent |
| 3 | Glioma, IDH1_R132H negative | n.m. | No match | n.a. | Unmethylated | Present |
| 4 | n.a. | n.m. | Plexus tumor | 0.61 | n.a. | n.a. |
| 5 | Glioma, IDH mutant | n.m. | Plexus tumor | 0.47 | n.a. | n.a. |

**Supplementary Table 2.** Sample IDs, final reported diagnoses, DNA concentrations, methylation class allocation, calibrated scores, MGMT promoter methylation status and copy number variation (CNV) information for the five samples removed from the STX study cohort due to failure on QC performed by the RnBeads algorithm. Not assessable (n.a.), not measurable (n.m.).

| **MGMT promoter methylation status** | **% (n)** |
| --- | --- |
| **Ascertainable** | **91.9% (205)** |
| methylated | 50.2% (103) |
| not methylated | 49.8% (102) |
| **Not ascertainable** | **8.1% (18)** |
| Σ | **100% (223)** |

**Supplementary Table 3.** Relative and absolute values of MGMT promoter ascertainability and status in STX gliomas.

| **CS** | **DNA amount not measurable** | **DNA amount >0<250ng** | **DNA amount >250ng** |
| --- | --- | --- | --- |
| **n** | 3 | 84 | 119 |
| **Mean** | 0.45 | 0.79 | 0.88 |
| **Standard deviation** | 0.19 | 0.23 | 0.18 |
| **Mean standard error** | 0.11 | 0.025 | 0.016 |
| **Mean CI lower** | -0.034 | 0.74 | 0.84 |
| **Mean CI upper** | 0.93 | 0.84 | 0.91 |
| **CS min** | 0.33 | 0.3 | 0.3 |
| **CS 25% quartile** | 0.33 | 0.63 | 0.84 |
| **CS median** | 0.34 | 0.91 | 0.96 |
| **CS 75% quartile** | 0.67 | 0.99 | 0.99 |
| **CS max** | 0.67 | 0.99 | 0.99 |
| **p values** |  |  |  |
| **1 vs. 2** | 0.1169 |  |  |
| **2 vs. 3** | *0.0262* |  |  |
| **1 vs. 3** | *0.0199* |  |  |

**Supplementary Table 4.** General statistical and quantile values of calibrated scores (CS) for DNA amount ranges “not measurable”, 2 “>0<250ng” and “>250ng”. P values Dunn’s test.

Calibrated scores: a) classifier methylation class match with H&E diagnosis

|  | **DNA amount not measurable** | **DNA amount >0<250ng** | **DNA amount >250ng** |
| --- | --- | --- | --- |
| **n** | 1 | 67 | 100 |
| **Mean** | 0.67 | 0.82 | 0.89 |
| **Standard deviation** | . | 0.21 | 0.16 |
| **Mean standard error** | . | 0.026 | 0.016 |
| **Mean CI lower** | . | 0.77 | 0.85 |
| **Mean CI upper** | . | 0.88 | 0.92 |
| **CS min** | 0.67 | 0.3 | 0.33 |
| **CS 25% quartile** | 0.67 | 0.72 | 0.85 |
| **CS median** | 0.67 | 0.94 | 0.97 |
| **CS 75% quartile** | 0.67 | 0.99 | 0.99 |
| **CS max** | 0.67 | 0.99 | 0.99 |
| **p values** |  |  |  |
| **1 vs. 2** | 1.0000 |  |  |
| **2 vs. 3** | 0.2057 |  |  |
| **1 vs. 3** | 0.6385 |  |  |

Calibrated scores: b) classifier upgrade compared to H&E diagnosis

|  | **DNA amount >0<250ng** | **DNA amount >250ng** |
| --- | --- | --- |
| **n** | 5 | 10 |
| **Mean** | 0.85 | 0.9 |
| **Standard deviation** | 0.22 | 0.16 |
| **Mean standard error** | 0.01 | 0.052 |
| **Mean CI lower** | 0.57 | 0.79 |
| **Mean CI upper** | 1.13 | 1.02 |
| **CS min** | 0.46 | 0.45 |
| **CS 25% quartile** | 0.68 | 0.9 |
| **CS median** | 0.91 | 0.96 |
| **CS 75% quartile** | 0.99 | 0.99 |
| **CS max** | 0.99 | 0.99 |
| **p value** |  |  |
| **2 vs. 3** | 0.8027 |  |

Calibrated scores: c) classifier downgrade compared to H&E diagnosis

|  | **DNA amount >0<250ng** | **DNA amount >250ng** |
| --- | --- | --- |
| **n** | 4 | 5 |
| **Mean** | 0.75 | 0.92 |
| **Standard deviation** | 0.25 | 0.13 |
| **Mean standard error** | 0.13 | 0.057 |
| **Mean CI lower** | 0.35 | 0.76 |
| **Mean CI upper** | 1.15 | 1.07 |
| **CS min** | 0.38 | 0.69 |
| **CS 25% quartile** | 0.48 | 0.82 |
| **CS median** | 0.85 | 0.97 |
| **CS 75% quartile** | 0.92 | 0.99 |
| **CS max** | 0.93 | 0.99 |
| **p value** |  |  |
| **2 vs. 3** | 0.1099 |  |

Calibrated score: d) Methylation class “no tumor”

|  | **DNA amount not measurable** | **DNA amount >0<250ng** | **DNA amount >250ng** |
| --- | --- | --- | --- |
| **n** | 1 | 7 | 3 |
| **Mean** | 0.33 | 0.5 | 0.52 |
| **Standard deviation** | . | 0.17 | 0.24 |
| **Mean standard error** | . | 0.064 | 0.14 |
| **Mean CI lower** | . | 0.34 | -0.067 |
| **Mean CI upper** | . | 0.66 | 1.11 |
| **CS min** | 0.33 | 0.31 | 0.3 |
| **CS 25% quartile** | 0.33 | 0.34 | 0.3 |
| **CS median** | 0.33 | 0.49 | 0.49 |
| **CS 75% quartile** | 0.33 | 0.66 | 0.77 |
| **CS max** | 0.33 | 0.79 | 0.77 |
| **p values** |  |  |  |
| **1 vs. 2** | 0.3799 |  |  |
| **2 vs. 3** | 1.0 |  |  |
| **1 vs. 3** | 1.0 |  |  |

**Supplementary Table 5.** General statistical and quantile values of calibrated scores (CS) for DNA amount ranges “not measurable”, “>0<250ng” and “>250ng” stratified by classifier result a) match with H&E diagnosis, b) upgrade compared to H&E diagnosis, c) downgrade compared to H&E diagnosis and d) “no tumor”. P values Dunn’s test.

| **split** | **response_variable** | **predictor_variable** | **value** | **logworth** |
| --- | --- | --- | --- | --- |
| **1** | CS >0.84 | DNA concentration | 1.21 | 1.84 |
| **1** | CS >0.84 | cancer cell proportion | 0.53 | 4.13 |
| **1** | CS > 0.9 | DNA concentration | 1.21 | 1.1 |
| **1** | CS > 0.9 | cancer cell proportion | 0.53 | 3.9 |
| **1** | BC > 0.9 | DNA concentration | 30.70 | 2.45 |
| **1** | BC > 0.9 | cancer cell proportion | 0.54 | 0.23 |
| **1** | BF > 0.9 | DNA concentration | 2.95 | 1.11 |
| **1** | BF > 0.9 | cancer cell proportion | 0.39 | 0.14 |
| **2** | CS >0.84 | DNA concentration | 1.21 | 1.38 |
| **2** | CS >0.84 | cancer cell proportion | 0.55 | 3.21 |
| **2** | CS > 0.9 | DNA concentration | 7.00 | 1.51 |
| **2** | CS > 0.9 | cancer cell proportion | 0.56 | 3 |
| **2** | BC > 0.9 | DNA concentration | 30.70 | 0.96 |
| **2** | BC > 0.9 | cancer cell proportion | 0.49 | 0.18 |
| **2** | BF > 0.9 | DNA concentration | 1.11 | 1.75 |
| **2** | BF > 0.9 | cancer cell proportion | 0.38 | 1.26 |
| **3** | CS >0.84 | DNA concentration | 5.52 | 1.21 |
| **3** | CS >0.84 | cancer cell proportion | 0.55 | 3.34 |
| **3** | CS > 0.9 | DNA concentration | 3.53 | 1.65 |
| **3** | CS > 0.9 | cancer cell proportion | 0.62 | 4.31 |
| **3** | BC > 0.9 | DNA concentration | 30.70 | 0.87 |
| **3** | BC > 0.9 | cancer cell proportion | 0.71 | 0.08 |
| **3** | BF > 0.9 | DNA concentration | 2.95 | 1.49 |
| **3** | BF > 0.9 | cancer cell proportion | 0.49 | 0.13 |
| **4** | CS >0.84 | DNA concentration | 1.21 | 1.06 |
| **4** | CS >0.84 | cancer cell proportion | 0.50 | 4.05 |
| **4** | CS > 0.9 | DNA concentration | 1.81 | 0.77 |
| **4** | CS > 0.9 | cancer cell proportion | 0.55 | 3.59 |
| **4** | BC > 0.9 | DNA concentration | 1.60 | 0.23 |
| **4** | BC > 0.9 | cancer cell proportion | 0.80 | 0.07 |
| **4** | BF > 0.9 | DNA concentration | 3.02 | 0.58 |
| **4** | BF > 0.9 | cancer cell proportion | 0.74 | 0.5 |
| **5** | CS >0.84 | DNA concentration | 5.12 | 1.8 |
| **5** | CS >0.84 | cancer cell proportion | 0.55 | 4.88 |
| **5** | CS > 0.9 | DNA concentration | 5.12 | 1.2 |
| **5** | CS > 0.9 | cancer cell proportion | 0.56 | 6.32 |
| **5** | BC > 0.9 | DNA concentration | 1.99 | 0.31 |
| **5** | BC > 0.9 | cancer cell proportion | 0.54 | 0.14 |
| **5** | BF > 0.9 | DNA concentration | 2.95 | 1.37 |
| **5** | BF > 0.9 | cancer cell proportion | 0.58 | 0.84 |
| **6** | CS >0.84 | DNA concentration | 1.21 | 2.58 |
| **6** | CS >0.84 | cancer cell proportion | 0.55 | 3.27 |
| **6** | CS > 0.9 | DNA concentration | 5.52 | 2.07 |
| **6** | CS > 0.9 | cancer cell proportion | 0.55 | 3.77 |
| **6** | BC > 0.9 | DNA concentration | 1.59 | 0.82 |
| **6** | BC > 0.9 | cancer cell proportion | 0.54 | 0.37 |
| **6** | BF > 0.9 | DNA concentration | 1.21 | 0.72 |
| **6** | BF > 0.9 | cancer cell proportion | 0.74 | 0.31 |
| **7** | CS >0.84 | DNA concentration | 8.84 | 0.5 |
| **7** | CS >0.84 | cancer cell proportion | 0.53 | 4.38 |
| **7** | CS > 0.9 | DNA concentration | 7.00 | 0.62 |
| **7** | CS > 0.9 | cancer cell proportion | 0.43 | 3.67 |
| **7** | BC > 0.9 | DNA concentration | 14.00 | 1.13 |
| **7** | BC > 0.9 | cancer cell proportion | 0.41 | 0.4 |
| **7** | BF > 0.9 | DNA concentration | 2.95 | 0.75 |
| **7** | BF > 0.9 | cancer cell proportion | 0.39 | 0.2 |
| **8** | CS >0.84 | DNA concentration | 5.52 | 2.26 |
| **8** | CS >0.84 | cancer cell proportion | 0.58 | 0.34 |
| **8** | CS > 0.9 | DNA concentration | 8.84 | 1.96 |
| **8** | CS > 0.9 | cancer cell proportion | 0.58 | 2.07 |
| **8** | BC > 0.9 | DNA concentration | 16.70 | 0.15 |
| **8** | BC > 0.9 | cancer cell proportion | 0.72 | 0.2 |
| **8** | BF > 0.9 | DNA concentration | 3.18 | 0.8 |
| **8** | BF > 0.9 | cancer cell proportion | 0.48 | 0.1 |
| **9** | CS >0.84 | DNA concentration | 1.21 | 2.41 |
| **9** | CS >0.84 | cancer cell proportion | 0.56 | 3.9 |
| **9** | CS > 0.9 | DNA concentration | 1.21 | 1.48 |
| **9** | CS > 0.9 | cancer cell proportion | 0.62 | 4.46 |
| **9** | BC > 0.9 | DNA concentration | 1.59 | 0.91 |
| **9** | BC > 0.9 | cancer cell proportion | 0.63 | 0.11 |
| **9** | BF > 0.9 | DNA concentration | 2.95 | 2.49 |
| **9** | BF > 0.9 | cancer cell proportion | 0.38 | 0.96 |
| **10** | CS >0.84 | DNA concentration | 5.12 | 1.28 |
| **10** | CS >0.84 | cancer cell proportion | 0.53 | 6.01 |
| **10** | CS > 0.9 | DNA concentration | 5.12 | 1.33 |
| **10** | CS > 0.9 | cancer cell proportion | 0.55 | 5.62 |
| **10** | BC > 0.9 | DNA concentration | 1.60 | 0.36 |
| **10** | BC > 0.9 | cancer cell proportion | 0.55 | 0.11 |
| **10** | BF > 0.9 | DNA concentration | 3.02 | 0.74 |
| **10** | BF > 0.9 | cancer cell proportion | 0.38 | 0.88 |
| **11** | CS >0.84 | DNA concentration | 1.21 | 1.84 |
| **11** | CS >0.84 | cancer cell proportion | 0.55 | 3.65 |
| **11** | CS > 0.9 | DNA concentration | 1.21 | 1.19 |
| **11** | CS > 0.9 | cancer cell proportion | 0.58 | 3.54 |
| **11** | BC > 0.9 | DNA concentration | 30.70 | 0.79 |
| **11** | BC > 0.9 | cancer cell proportion | 0.41 | 0.44 |
| **11** | BF > 0.9 | DNA concentration | 2.95 | 3.17 |
| **11** | BF > 0.9 | cancer cell proportion | 0.60 | 1.13 |
| **12** | CS >0.84 | DNA concentration | 1.21 | 1.17 |
| **12** | CS >0.84 | cancer cell proportion | 0.63 | 2.57 |
| **12** | CS > 0.9 | DNA concentration | 1.21 | 0.75 |
| **12** | CS > 0.9 | cancer cell proportion | 0.62 | 3.08 |
| **12** | BC > 0.9 | DNA concentration | 1.59 | 0.24 |
| **12** | BC > 0.9 | cancer cell proportion | 0.67 | 1.02 |
| **12** | BF > 0.9 | DNA concentration | 2.95 | 1.13 |
| **12** | BF > 0.9 | cancer cell proportion | 0.62 | 0.73 |
| **13** | CS >0.84 | DNA concentration | 1.21 | 1.97 |
| **13** | CS >0.84 | cancer cell proportion | 0.55 | 4.11 |
| **13** | CS > 0.9 | DNA concentration | 1.21 | 1.23 |
| **13** | CS > 0.9 | cancer cell proportion | 0.55 | 6.29 |
| **13** | BC > 0.9 | DNA concentration | 1.33 | 0.57 |
| **13** | BC > 0.9 | cancer cell proportion | 0.52 | 0.24 |
| **13** | BF > 0.9 | DNA concentration | 2.95 | 1.81 |
| **13** | BF > 0.9 | cancer cell proportion | 0.48 | 0.21 |
| **14** | CS >0.84 | DNA concentration | 1.21 | 1.32 |
| **14** | CS >0.84 | cancer cell proportion | 0.55 | 4.04 |
| **14** | CS > 0.9 | DNA concentration | 29.20 | 0.99 |
| **14** | CS > 0.9 | cancer cell proportion | 0.55 | 4.88 |
| **14** | BC > 0.9 | DNA concentration | 30.70 | 0.65 |
| **14** | BC > 0.9 | cancer cell proportion | 0.75 | 0.11 |
| **14** | BF > 0.9 | DNA concentration | 1.21 | 0.51 |
| **14** | BF > 0.9 | cancer cell proportion | 0.58 | 0.45 |
| **15** | CS >0.84 | DNA concentration | 5.52 | 1.39 |
| **15** | CS >0.84 | cancer cell proportion | 0.43 | 4.36 |
| **15** | CS > 0.9 | DNA concentration | 8.84 | 1.02 |
| **15** | CS > 0.9 | cancer cell proportion | 0.65 | 6.36 |
| **15** | BC > 0.9 | DNA concentration | 32.70 | 0.42 |
| **15** | BC > 0.9 | cancer cell proportion | 0.65 | 0.31 |
| **15** | BF > 0.9 | DNA concentration | 2.04 | 1.36 |
| **15** | BF > 0.9 | cancer cell proportion | 0.39 | 0.96 |
| **16** | CS >0.84 | DNA concentration | 1.21 | 2.67 |
| **16** | CS >0.84 | cancer cell proportion | 0.53 | 4.83 |
| **16** | CS > 0.9 | DNA concentration | 1.59 | 2.14 |
| **16** | CS > 0.9 | cancer cell proportion | 0.58 | 3.31 |
| **16** | BC > 0.9 | DNA concentration | 30.70 | 2.06 |
| **16** | BC > 0.9 | cancer cell proportion | 0.55 | 0.12 |
| **16** | BF > 0.9 | DNA concentration | 1.21 | 1.04 |
| **16** | BF > 0.9 | cancer cell proportion | 0.58 | 0.24 |
| **17** | CS >0.84 | DNA concentration | 1.21 | 1.25 |
| **17** | CS >0.84 | cancer cell proportion | 0.43 | 4.74 |
| **17** | CS > 0.9 | DNA concentration | 1.59 | 0.97 |
| **17** | CS > 0.9 | cancer cell proportion | 0.43 | 4.32 |
| **17** | BC > 0.9 | DNA concentration | 13.60 | 0.65 |
| **17** | BC > 0.9 | cancer cell proportion | 0.80 | 0.13 |
| **17** | BF > 0.9 | DNA concentration | 2.95 | 0.81 |
| **17** | BF > 0.9 | cancer cell proportion | 0.38 | 1.15 |
| **18** | CS >0.84 | DNA concentration | 1.21 | 0.93 |
| **18** | CS >0.84 | cancer cell proportion | 0.44 | 4.58 |
| **18** | CS > 0.9 | DNA concentration | 1.33 | 0.67 |
| **18** | CS > 0.9 | cancer cell proportion | 0.44 | 3.16 |
| **18** | BC > 0.9 | DNA concentration | 1.59 | 1.22 |
| **18** | BC > 0.9 | cancer cell proportion | 0.65 | 0.13 |
| **18** | BF > 0.9 | DNA concentration | 51.20 | 0.5 |
| **18** | BF > 0.9 | cancer cell proportion | 0.39 | 0.88 |
| **19** | CS >0.84 | DNA concentration | 1.21 | 1.73 |
| **19** | CS >0.84 | cancer cell proportion | 0.43 | 3.81 |
| **19** | CS > 0.9 | DNA concentration | 1.81 | 1.73 |
| **19** | CS > 0.9 | cancer cell proportion | 0.43 | 3.98 |
| **19** | BC > 0.9 | DNA concentration | 3.03 | 1.76 |
| **19** | BC > 0.9 | cancer cell proportion | 0.55 | 0.16 |
| **19** | BF > 0.9 | DNA concentration | 2.95 | 2.32 |
| **19** | BF > 0.9 | cancer cell proportion | 0.38 | 1.15 |
| **20** | CS >0.84 | DNA concentration | 1.59 | 1.74 |
| **20** | CS >0.84 | cancer cell proportion | 0.56 | 4.85 |
| **20** | CS > 0.9 | DNA concentration | 1.88 | 1.13 |
| **20** | CS > 0.9 | cancer cell proportion | 0.56 | 6.22 |
| **20** | BC > 0.9 | DNA concentration | 13.60 | 0.79 |
| **20** | BC > 0.9 | cancer cell proportion | 0.61 | 0.05 |
| **20** | BF > 0.9 | DNA concentration | 30.70 | 0.41 |
| **20** | BF > 0.9 | cancer cell proportion | 0.74 | 0.37 |

**Supplementary Table 6.** Subsampling stability analysis, 20 iterations, drawing of a random subset of 70% of cases without replacement for re-fitting of the recursive partitioning model. Raw values corresponding to Supplementary Figure 4. BC: Bethesda brain tumor classifier, class; BF: Bethesda brain tumor classifier, family; CS: Heidelberg brain tumor classifier, calibrated score.

**Supplementary Excel File.**
